# Revisiting nerve nets: functional organization and evolutionary implications

**DOI:** 10.1038/s44318-026-00879-w

**Published:** 2026-07-23

**Authors:** Kei Jokura, Gáspár Jékely

**Affiliations:** 1 Exploratory Research Center on Life and Living Systems (ExCELLS), Okazaki, 444-8787 Japan; 2 National Institute for Basic Biology (NIBB), Okazaki, 444-8585 Japan; 3 Heidelberg University, Centre for Organismal Studies (COS), Heidelberg, 69120 Germany

**Keywords:** Evolution & Ecology, Neuroscience

## Abstract

Nerve nets—characterised by scattered neurons and mesh-like projections—represent a widespread organisation of nervous tissue. Nerve nets have uniquely diffuse conductivity and an apparent simplicity that features in many theories on the evolution of nervous systems. Here we review the anatomical and functional organisation of nerve nets and revisit evolutionary theories in light of new information. We discuss how transgenesis and volume electron microscopy begin to reveal the fine-scale cellular organisation of nerve nets. On the functional level, we describe how excitation can spread within nerve nets and how non-synaptic volume transmission enable multi-layered signalling, driving complex behaviours. As a convergent solution to tissue-scale coordination, we introduce ctenophores, where syncytial nerve nets implement distributed control. We argue that nerve nets are not primitive remnants in some animals or only historical preludes but represent an enduring organisational principle for animal coordination with many unique advantages.

## Abstract (web summary)

Far from primitive, nerve nets are sophisticated distributed neural systems.

## Graphical abstract

**Figure:**
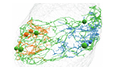

## Introduction

The term ‘nerve net’ refers to an overall anatomical organisation of nervous tissue with a regular mesh-like arrangement of neuronal processes. Nerve nets lack an obvious polarity and do not show a bundling of neurites into cords or the clustering of neuronal somata into ganglia.

**Figure 1:**
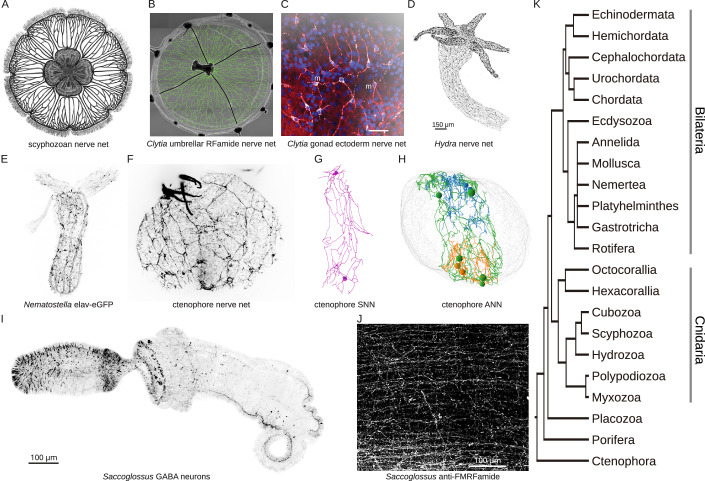
Examples of nerve nets. (**A**) Schematic of the nerve net of a schyphozoan medusa, after Romanes (1885). (**B**) RFamide-positive nerve net in the medusa of the hydrozoan Clytia hemispaerica (manual segmentation), after (Weissbourd et al, 2021). (**C**) Morphology of the gonad ectodermal nerve net in Clytia stained with an antibody against the neuropeptide PRPamide. Image shows a confocal plane at the base of the ectoderm. The PRGamide antibody (white) labels peptidergic vesicles in the somata and the projections of the neurons. The anti-α-tubulin staining (red) highlights the microtubule bundles in the basal projections. Scale bar 20 µm, after Quiroga Artigas et al (2020). (**D**) Hydra upper body column including the hypostome and tentacles stained with a pan-neuronal antibody, after Keramidioti et al (2024). (**E**) Transgenic labelling of the elav+ nervous system in the anthozoan Nematostella vectensis. Image, courtesy of Aissam Ikmi. (**F**) Nerve net of a ctenophore immunostained for tubulin. (**G**) Detail of the subepithelial nerve net (SNN) of a 1-day-old Mnemiopsis cydippid, reconstructed by volume EM. Adapted from Burkhardt et al (2023). (**H**) 3D reconstruction of the aboral nerve net in Mnemiopsis, after Jokura et al (2025). (**I**) GABA neurons in the hemichordate Saccoglossus kowalevskii. (**J**) Adult proboscis plexus of a Saccoglossus late juvenile labelled with an antibody against FMRFamide. Anterior to the left, after Andrade López et al (2023). (**K**) Phylogenetic overview of metazoan lineages, adapted from Yañez-Guerra et al (2022).

Nerve nets are found across multiple animal lineages, including cnidarians (Fig. 1A–E), ctenophores (Fig. 1F–H), acoel flatworms, hemichordates (Fig. 1I,J) and many other groups (Hejnol and Rentzsch, 2015) (see Fig. 1K for a phylogenetic overview of major animal lineages). Classical anatomical studies often failed to reveal the neurite architecture and synaptic connectivity of individual neurons within nerve nets. Because of this, the broad term ‘nerve net’ masks a rich diversity of underlying neuroanatomies, synaptic wiring and functions.

In the first part of this review, we give an overview of classical and recent work on the cellular and synaptic organisation of nerve nets. We discuss how new transgenic technologies and tissue-scale volume electron microscopy (vEM) start to give a clearer, sometime synapse-level view of the organisation of nerve nets. As a starting point, we conceptualise a random nerve net and then show how real biological nerve nets deviate from such a null pattern. We show that the neuronal architectures of nerve nets can be vastly different, despite their superficial resemblance. We also introduce classical behavioural and electrophysiological experiments and more recent calcium imaging and modelling studies and discuss what they have revealed about the dynamics of nerve nets.

In the second part, we consider the evolution of nerve nets and the origin of centralised nervous systems. Nerve nets have featured prominently in theories about the evolution of nervous systems in two main contexts. One context is transition scenarios on the origin of nervous systems where nerve-nets are often hypothesized to have provided a primitive form of nervous integration (Keijzer et al, 2013). The other context is theories on the evolution of nervous system centralisation (Holland, 2003; Northcutt, 2012). In light of new functional and anatomical evidence on nerve nets, we revisit these theories. We argue that nerve nets often do not represent a primitive, ancestral state but advanced distributed neural architectures to coordinate and control certain body forms and locomotion strategies. In this sense nerve nets represent a successful and often highly evolved organisation in extant animals. Finally, we argue that centralisation into ganglia and nerve bundles may have deeper evolutionary origins than often appreciated.

## Nerve nets—conceptualisation and definitions

A widespread perception of nerve nets is that they are diffuse networks of neurons with random projections and connectivity. According to the definition of Hejnol and Rentzsch “a basic nerve net consists of an irregular arrangement of neurites” for the formation of which the “random outgrowth of neuronal processes in a permissive environment might be sufficient” (Hejnol and Rentzsch, 2015). Further paraphrased quotes assuming a random or ‘diffuse’ organisation of nerve nets are cited in Satterlie (2011).

The terminology to describe nerve nets is sometimes confusing and terms like ‘plexus’, ‘nerve net’ or ‘diffuse nervous system’ are used interchangeably. We will follow the terminology of Richter et al (2010). They defined a ‘plexus’ as a cluster of neurons that consists of neuronal somata and their neurites arranged in a planar reticular pattern. A nerve net is a form of intraepidermal plexus formed by dispersed neurons that are “connected either by synaptic contact or fusion in such a way as to permit the diffuse conduction of excitation”. A ganglion is, in turn, a cluster of neurons with neuronal somata concentrated at the surface and neurites concentrated in the centre to form the neuropil.

**Figure 2:**
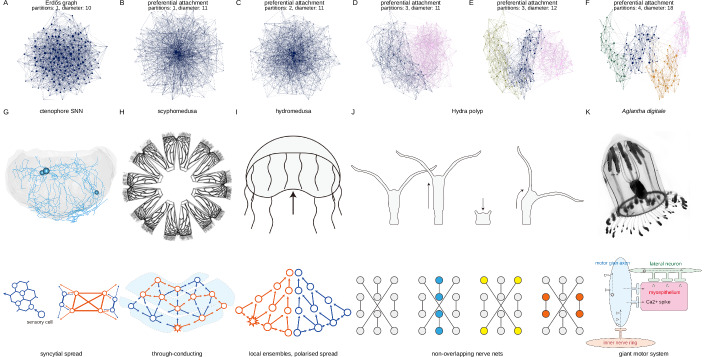
Cellular organisation. (**A**) Erdős–Rényi-type random graph with 200 nodes and 1990 connections. (**B**–**F**) Preferential-attachment graphs with 200 nodes and 1990 connections and a decreasing ageing exponent (from 0 to −2), resulting in increasingly modular graphs. (**G**) Syncytial nerve net of the ctenophore Mnemiopsis (top; after Jokura et al (2025)) and schematic of activity spread in a syncytium (bottom). (**H**) Schematic of an incision experiment carried out by Romanes on the scyphosoan medusa Aurelia, after Romanes (1885). The umbrella is through-conducting, mediated in part by epithelial conductivity. (**I**) Schematic of the localised spread of activity in a hydrozoan nerve net (after Weissbourd et al (2021)) and (**J**) in Hydra (after Dupre and Yuste (2017)), where different non-overlapping nerve nets mediate distinct behaviours. (**K**) The hydromedusa Aglantha digitale with its giant motor system shows a centralised arrangement. Image by Erling Svensen: https://www.artsdatabanken.no/Pages/F21737. Schematic adapted from Meech (2015).

So far a quantitative, formal definition of nerve nets is lacking. We will use graph theory to develop a clearer conceptual framework for nerve nets. We start with the definition of a random graph (Erdős and Rényi, 1959). In an Erdős–Rényi-type random graph *G(n,M)*, there are *n* nodes that are connected with the same probability by *M* edges. In a biological nerve net, this would translate to *M* randomly formed synaptic connections (edges) between *n* neurons (nodes) (Fig. 2A). We could add that in a random biological nerve net, neurons would be randomly scattered in a tissue with randomly-projecting (‘irregular’) neurites that conduct both away from and towards the soma. We call this the null hypothesis for nerve nets (Fig. 2A). This framework will guide the following discussion of the diversity of nerve nets. Below, we examine nerve nets based on four characteristics, (i) the distribution of neuronal cell bodies, (ii) cell-type diversity, (iii) projection patterns, and (iv) synaptic connectivity. Ultimately, we may be able to place nerve nets along a gradient from the most diffuse towards the more specialised, partitioned or centralised (Fig. 2A–F).

### Cellular organisation—distribution of neuronal somata

First we examine the distribution of neuronal somata in nerve nets. This distribution is of course never random because the patterning of neural progenitors and neuronal differentiation happen in the regulatory environment of polarised embryos and larvae (Hartenstein and Stollewerk, 2015). Molecular studies revealed both spatial and temporal patterning mechanisms (Havrilak et al, 2025; Faltine-Gonzalez et al, 2023). For example, in cnidarians, anterio-posterior patterning and planar polarity are regulated by Wnt signalling (Momose et al, 2012; Uveira et al, 2025; Noro et al, 2019; Kusserow et al, 2005). Different neuron populations differentiate along the anterior-posterior axis specified by a conserved anteroposterior axial programme (Noro et al, 2019; Faltine-Gonzalez et al, 2023). While several neuronal markers (e.g. neuropeptides) give staining patterns that are described as ‘salt and pepper’ (Havrilak et al, 2025), these patterns are also not random upon closer examination (Rentzsch et al, 2016). Rather neurons tile a surface uniformly with individual neurons often following anatomical landmarks, e.g. the position of mesenteries (longitudinal endodermal infoldings) in anthozoan polyps (Havrilak et al, 2017; Tournière et al, 2020) occupying distinct antero-posterior domains (Piraino et al, 2011; Ramon-Mateu et al, 2025). In medusae of the hydrozoan *Clytia*, RFamide-positive neurons that constitute a subset of the nerve-net neurons are radially oriented, running along radial muscle fibres (Weissbourd et al, 2021).

Cnidarian nerve nets can also be partially centralised. Immunolabellings for RFamide neuropeptides revealed nerve bundles and plexi across cnidarian species including the clustering of sensory neurons around the mouth (Grimmelikhuijzen, 1985; Eichinger and Satterlie, 2014; Satterlie, 2011).

In hemichordates—bilaterian animals with a diffuse nerve plexus and belonging to the ambulacraria—neuronal cell types show clear regionalisation, despite the diffuse appearance of the nervous system by pan-neural markers (Fig. 1I, J). In *Saccoglossus kowalevskii*, a recent detailed anatomical study revealed a non-random distribution of many neuronal cell types with anteroposterior and dorsoventral regionalisation (Andrade López et al, 2023). In the acoel flatworm *Hofstenia miamia*, neuronal markers reveal a layered organisation with distinct neural types occupying distinct territories (Hulett et al, 2020).

### Cellular organisation—cell-type diversity

Random nerve nets of the simplest hypothetical case would only consist of one neuron type. We know that this is not the case as there is ample evidence for molecular, morphological and functional specialisations.

In the small polyp *Hydra vulgaris*, the nerve net is formed by at least 11 transcriptionally different neuron types (Siebert et al, 2019; Morris Little et al, 2025). The endodermal nerve net recently reconstructed from a small *Hydra* consists of 20 cells but is formed by at least five morphologically distinct neuronal cell types, including sensory cells (Zhang et al, 2025). These molecular and morphological differences likely equal different functionality. For example, some of the endodermal neurons are sensory, others neurosecretory or ganglion(interneuron)-like. Different neuron types also express different combinations of neuropeptides indicating differential contributions to chemical signalling.

In the sea anemone *Nematostella vectensis*, both the ectodermal and endodermal nerve nets consist of distinct molecular territories and neuron types (Marlow et al, 2009), forming at least 18 transcriptional types (Cole et al, 2024). Similar diversity characterises the simplest bilaterians with a nerve net, the xenacoelomorpha. The nervous system of the acoel *Isodiametra pulchra* has 10 neuronal clusters (Duruz et al, 2020), and similar numbers were reported for *Xenoturbella bocki* (Robertson et al, 2024).

### Cellular organisation—neuronal projections

The null hypothesis of a random net posits that neurons have random, non-polarised projections. In stark contrast to this, neurite outgrowth in many nerve nets is in stereotyped direction and with fixed polarity. Projections often follow anatomical landmarks such as muscle fibres, mesenteries or ciliary structures.

Observations of neural development in a transgenic line of the anthozoan *Nematostella vectensis* revealed that ectodermal neurons have a preferential orientation of neurite outgrowth (Stone et al, 2020; Stone et al, 2025). During larval development, early-born neurons grow their neurites towards the aboral region, whereas later-born neurons develop neurites that grow transversely (Stone et al, 2025). Live imaging of transgenic *Nematostella* revealed that some branched neurons have a mixed microtubule polarity suggesting the presence of distinct axonal and dendritic branches (Rolls and Jegla, 2015; Stone et al, 2020; Stone et al, 2025). In planula larvae of the hydrozoan *Podocoryne carnea*, a subset of the nervous system (positive for tyrosinated tubulin) develops neurites oriented perpendicular to the anterior-posterior axis. Later-developing neurites instead project from anterior to posterior (Gröger and Schmid, 2001). In adult hydrozoans, bipolar ganglion cells orient parallel to the longitudinal muscles (Stokes, 1974). Even in the highly diffuse nerve net of the acoel *Hofstenia*, nerve-net axons are closely aligned with peripheral muscle fibres (Hulett et al, 2020).

Ctenophores possess a polygonial subepithelial nerve net (SNN) and a distinct aboral nerve net (ANN). The SNN covers the entire body and pharynx surface, the ANN covers the centre of the aboral organ (Fig. 1F–H). A detailed quantification of the geometrical organisation of the SNN across different body regions and sizes in *Pleurobrachia pileus* revealed specific organizational rules. The network shows structural directionality, regional specialisations, stereotypy across animals, and a uniform size and shape of the nerve-net polygons across animals of different sizes (Courtney et al, 2022). Polarisation is also observed in the SNN of other ctenophores. In the oral region of *Vallicula multiformis*, a benthic ctenophore, the nerve net extends radially and distally from the mouth opening (Mohri and Watanabe, 2024).

The hemichordate *S. kowalevskii* has a well-organised plexus in many parts of the body. Dye injections revealed orderly projection that are different along the apicobasal axis in the plexus. Labelling subsets of neurons by neurotransmitter antibodies or transgenic constructs highlighted many polarised neuronal architectures and orderly projections, including serotonergic and GABAergic neurons (Andrade López et al, 2023).

The most diffuse nerve nets are found within the ectodermal and endodermal nerve nets of hydrozoans (Cnidarian) (Horridge, 1956; Passano and Passano, 1971; Lin et al, 2001; Keramidioti et al, 2024) (Fig. 2H,I). In scyphomedusae, the nerve net consists of sensory cells as well as bi- and multipolar neurons that can propagate action potentials along both directions in a neurite (Horridge, 1956; Anderson and Schwab, 1981; Anderson and Schwab, 1983; Satterlie, 2002b). The ectodermal and endodermal nerve nets of the small freshwater hydrozoan *Hydra* are also diffuse, although the ecto- and endodermal nets differ in anatomy and function.

A similarly diffuse organisation characterises members of the Xenacoelomorpha. These bilaterian animals are fully ciliated and flatworm-like in appearance, but are only distantly related to the Platyhelminthes flatworms. They have a brain and longitudinal neurite bundles and a diffuse periferal nerve net (Børve and Hejnol, 2014). The most diffuse arrangement is found within the nemertodermatids and xenoturbellids. These animals have a diffuse basiepidermal nerve plexus consisting of ciliated sensory neurons with their somata in the middle of the epidermis and bipolar or multipolar interneurons located more basally (Raikova et al, 2000a; Raikova et al, 2000b; Raikova et al, 2004). Among the Xenacoelomorpha, the acoel *Hofstenia miamia* also shows a highly diffuse organisation with a layered peripheral nerve net (Hulett et al, 2020). This diffuse organisation is best interpreted as a derived trait within the group (Martinez et al, 2024). Little is known about the fine-scale organisation and function of these nerve nets.

### Cellular organisation—connectivity

As the fourth organisational metric, we examine connectivity. Most biological nerve nets deviate from a null pattern and show selective (non-random) and polarised connectivity.

**Figure 3:**
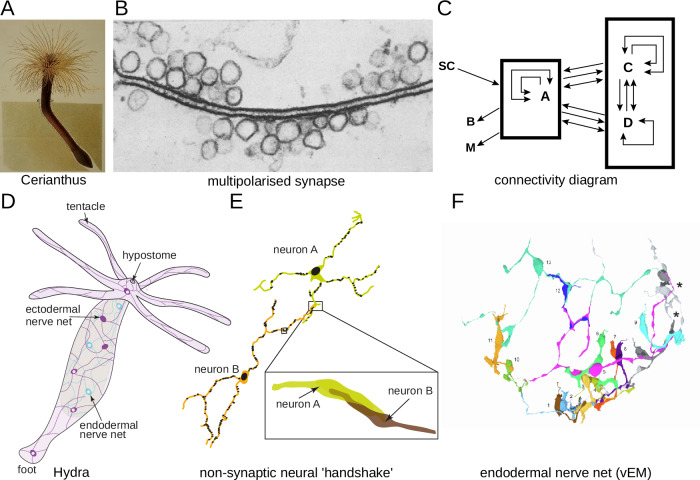
EM reconstruction of nerve nets. (**A**) Cerianthus, a cerianthid cnidarian, from (von Heider, 1879). (**B**) EM image of a bidirectional (multipolarized) synapse in Cerianthus. (**C**) Connectivity diagram of Ceriantheopsis americanus based on sparse EM sections, after Peteya (1973). (**D**) Schematic of Hydra with ectodermal and endodermal nerve nets, after Kerbl and Jékely (2025). (**E**) Schematic of a neuronal handshake between two endodermal neurons. (**F**) Volume EM reconstruction of the endodermal nerve net in a small Hydra. Adapted from Zhang et al (2025).

In *Hydra*, the nerve net forms many polarised synaptic contacts on postsynaptic partners (Westfall et al, 1971a). In the endodermal nerve net, synapses are lacking, but neurons connect via specialised protrusions that interdigitate with partner neurites. These structures—termed “neuronal handshakes”—only form between immediate neighbours (Zhang et al, 2025) (Fig. 3D–F). Similarly, in the gonad of *Clytia hemisphaerica*, the peptidergic neurons that constitute the nerve net form a regular lattice with neurites reaching to neighbouring cells (Quiroga Artigas et al, 2018). These nerve nets thus form non-random lattices or ‘simplicial complexes’ rather than random nets (in a random net neurons would connect to any other neuron not only their immediate neighbours).

Synapses in the ctenophore nerve net are likewise polarised and non-random (Hernandez-Nicaise, 1974; Jokura et al, 2025). In the larval subepithelial nerve net (SNN), conventional chemical synapses are formed specifically onto defined sensory-cell types, while synapses between SNN neurons themselves are rarely observed (Hernandez-Nicaise, 1973, 1974; Burkhardt et al, 2023). This pattern indicates a biased, feed-forward organisation rather than diffuse or random connectivity. In *Beroë*, the giant SNN at the lips forms multi-effector synapses onto both muscle and adhesive epithelium, establishing a hard-wired circuit that enables rapid mouth opening during prey capture (Tamm and Tamm, 1993; Tamm and Tamm, 1995; Norekian and Moroz, 2019). Likewise, neurons of the aboral nerve net (ANN) show selective, directional connectivity. They synapse on the balancer cells but not on the adjacent lamellate-body photoreceptors (Jokura et al, 2025). Inputs onto ANN neurons are rare but also very specific (only from bridge cells).

EM studies thus revealed a synaptic architecture of nerve nets that often strongly deviates from random, is polarised and has specific connectivity.

One exception is bidirectional synapses observed in cnidarians (Anderson and Schwab, 1981; Anderson, 1985) and ctenophores (Westfall, 1996). In such cases, each partner serves both as pre- and post-synaptic neuron. In the jellyfish *Cyanea* bidirectional synapses are physiologically unpolarised and conduct equally well in either direction (Anderson, 1985). In the cerianthid *Ceriantheopsis americanus* only an estimated 3% of interneural synapses are bidirectional (or ‘multipolarized’) (Peteya, 1973).

## Connectomics of nerve nets

Anatomical work and small-scale EM studies discussed above thus revealed the polarised nature of nerve nets with specific synaptic connectivity. A fascinating challenge is then to map nerve-net connectivity at the synaptic level. Such wiring diagrams or connectomes across animals with various degrees of centralisation could give a quantitative metric for comparisons (Hartenstein et al, 2025). Connectomics could also help us interpret behavioural and activity-imaging results and inform more precise modelling. Synaptic resolution requires electron microscopy (EM).

In a pioneering study, Peteya was able to infer synaptic connectivity of the ectodermal nerve net of the anthozoan cerianthid *Ceriantheopsis americanus* (Peteya, 1973) (Fig. 3A–C). In single EM sections, he identified sensory neurons and different types of interneurons, distinguishable by axon diameter. By systematic analysis of a large number of neurite profiles and the synapses among them, he obtained a connectivity diagram. The most common neurons were multipolar neurons (type A) with short branches of ~1 μm. These receive sensory input and synapse on muscles. The second and less common type is a small bipolar neuron (C type) with 4–6 μm diameter axons. The third type (D type) has axons with a diamater of 10–20 μm. Based on this, Peteya proposed that the cerianthid nervous system is organized into two conducting systems. One is composed of the short-range type A neurons that form a “slow-conducting system” mediating slow behaviours. The second is a “through-conducting-system” formed by long-range thick axons mediating rapid withdrawal into the tube, in agreement with physiological measurements (Horridge, 1958).

Recently, more comprehensive vEM resources of nerve nets have become available, one for *Hydra vulgaris* (Zhang et al, 2025) and two volumes for the ctenophore *Mnemiopsis leidyi* (Burkhardt et al, 2023; Jokura et al, 2025).

In *Hydra*, the endodermal nerve net of a small animal has been reconstructed. This nerve net is formed of 20 neurons, wherein neurons only connect to their immediate neighbours. Surprisingly, no classical chemical synapses could be identified in this dataset. Instead, neurites of adjacent neurons formed protrusions, which interdigitated with partner neurites. These neural handshakes were devoid of vesicles indicating that they do not function as classical synapses (Fig. 3D–F). It remains to be determined if these handshakes contain gap junctions, as seems likely (Keramidioti et al, 2024).

**Figure 4:**
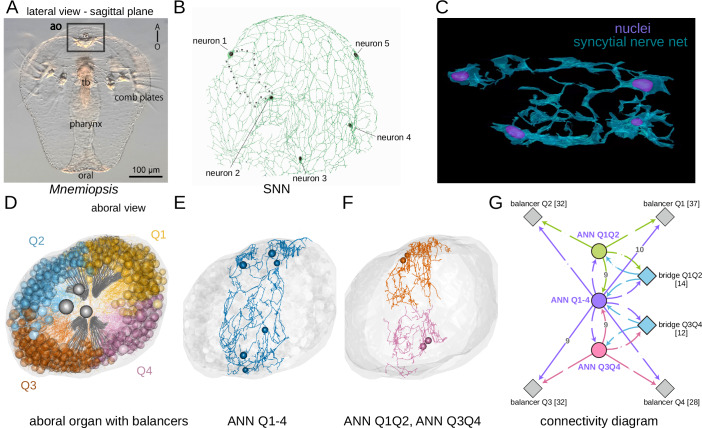
Connectome of the ctenophore aboral nerve net. (**A**) A Mnemiopsis leidyi cydippid larva. (**B**) 3D reconstructions of the Mnemiopsis subepithelial nerve net (SNN) in a 1-day-old cydippid. Adapted from Burkhardt et al (2023) and Ferraioli et al (2026). (**C**) 3D reconstruction of a segment of the aboral nerve net in Mnemiopsis. (**D**) 3D skeletonised reconstruction of the Mnemiopsis aboral organ with the four balancers. (**E**) Morphological rendering of the large ANN neuron (Q1–4). (**F**) Morhphological rendering of the two smaller ANN neurons (Q1Q2, Q3Q4). (**G**) Complete synaptic wiring diagram of the gravity-sensing circuit in the Mnemiopsis 5-day-old cydippid aboral organ. Cells belonging to the same type are shown as diamonds, with the number of cells shown in square brackets. The number of synapses is shown on the arrows. Adapted from Jokura et al (2025).

A vEM study of cydippid larvae of the ctenophore *Mnemiopsis leidyi* revealed a unique syncytial or reticulated architecture of the subepithelial nerve net (SNN). This syncytial nerve net covers the entire body and pharynx surface (Fig. 4A,B). Dye injections suggest that the entire nerve net is one large, multi-nucleate syncytium (Burkhardt et al, 2023).

A comprehensive nerve-net connectome so far only exist for the ctenophore aboral organ (Fig. 4A,D). The aboral nerve-net (ANN) in the 5-day-old cydippid stage is structurally distinct from the body wall SNN neurons (Burkhardt et al, 2023) (Fig. 4B). ANN neurons do not have a “beads-on-a-string” organisation like the SNN but form more uniform reticular processes (Fig. 4C) (Jokura et al, 2025).

The ANN consists of three syncytial neurons (Jokura et al, 2025), a large one spanning all four quadrants of the aboral organ and two smaller ones covering two adjacent quadrants each (Fig. 4E,F). The ANN synapses on the mechanosensory balancer cells and other cells and only receives synaptic input from the so called bridge cells. There are thus no synaptic inputs from sensory cells (unlike in the SNN (Burkhardt et al, 2023)) and no connections to motor effectors (muscles and comb plates) (Fig. 4G). The connectome and the high-speed imaging of balancer cilia suggest that the ANN functions to coordinate the activity of balancer cells, rather than as a sensory-motor-command network connecting to locomotor effectors (Jokura et al, 2025). The organisation of this nerve net is rather modular.

More vEM work will be needed in an increasing number of species to quantitatively compare nerve-net circuits and place them in the gradient from random to centralised (Fig. 2A–F).

## Spread of activity within nerve nets

In jellyfish, the “passage of the contraction-waves is dependent on the functional activity of the nervous plexus” as described by the classic work of G. J. Romanes (Romanes, 1885; Passano, 1965; Satterlie, 2002a). Contractions are induced by a pacemaker system that is localised to the rhopalia in scyphomedusae and to an inner marginal nerve ring in hydromedusae (Romanes, 1885).

The motor nerve net in scyphomedusae is “through-conducting”, which means that activity originating in one part is transmitted throughout the entire system (Satterlie, 2002a). This spread is non-polarised as first shown by Romanes in a series of elegant incision experiments (Romanes, 1885) (Fig. 2H). When he incised the umbrella in complex patterns, he found that the transmission of the excitation could pass around the incisions, showing diffuse and non-polarised spread. However, later Mackie, Passano and others showed that hydroids have neuroid conduction via epithelia, which can explain the results of the incisions (Mackie, 1970; Passano, 1973).

In jellyfish, bidirectional, non-polarised synapses were described by EM (Anderson and Schwab, 1981) and such synapses conduct equally well in either direction as shown by electrophysiology in the scyphozoan jellyfish *Cyanea* (Anderson, 1985). Furthermore, the bipolar neurons within scyphozoan nerve nets can propagate action potentials in either direction (Anderson and Schwab, 1981; Anderson and Schwab, 1983). These features underlie the through-conduction ability of scyphozoan nerve nets (resembling graphs in Fig. 2A–C). A multi-scale mathematical model developed for the jellyfish *Aurelia aurita* constrained by experimental studies could capture the dynamics of activity spread and how it drives whole-animal swimming (Pallasdies et al, 2019).

Electrical recordings from hydromedusae found that their nerve nets show more regional autonomy than in scyphomedusae (Passano, 1965; Satterlie, 2002b). This regionalisation was recently confirmed by calcium imaging experiments (Weissbourd et al, 2021; Cunningham et al, 2024). The use of a genetically-encoded calcium indicator GCaMP6s has enabled organism-wide imaging of nerve-net activation in the small hydrozoan jellyfish *Clytia hemisphaerica* (Weissbourd et al, 2021). This study was enabled by the transgenic labelling of RFamide-positive neurons that form a sub-net in the umbrella. RFamide neurons activate during episodes of infolding of the umbrella margin, but not during swimming. Activity spreads at the sites of infolding, in radially oriented localised patches. The chemogenetic ablation of these neurons eliminated folding behaviour. RFamide-positive neurons thus show regionalised synchronous activation in local ensembles driving infolding. These co-activating ensembles vary in size and do not show clear borders, with neighbouring neurons more likely to activate together, revealing distance-dependent coupling. Activity starts from the nerve rings at the margin and spreads into the nerve net and towards the mouth. An incision in the subumbrella can block the centripetal spread revealing its polarity and that the RFamide-positive nerve net is not through-conducting (Weissbourd et al, 2021) (resembling graphs in Fig. 2D,E).

A minimal excitable-sheet model could explain how global coordination can arise in the *Clytia* nerve net without long axons or nerve bundles (Weissbourd et al, 2021). By only using local connectivity, the model produced activity patterns that closely matched the calcium imaging data. Large-scale coordination thus emerges from local excitation spreading through a densely tiled nerve net, without requiring centralized structures.

In *Hydra* polyps, whole-body calcium imaging showed that there are at least three distinct nerve nets, each with ensemble-level synchrony. These ensembles activate near-simultaneously and broadly through the meshwork (Dupre and Yuste, 2017; Noro et al, 2019). During regeneration, the ensembles reform and resynchronize, indicating an intrinsic capacity to rebuild propagating patterns (Lovas and Yuste, 2021). The activation of the separate nerve nets correlates with different behaviours, including elongation, contraction or bending. For example, the ecodermal nerve net, but not the endodermal net, activates during fast body-column contractions (Dupre and Yuste, 2017) (Fig. 2J). Moreover, reciprocal inhibition between major ensembles such as RP1 (rhythmical potential) and CB (contraction burst) neurons produces alternating, network-wide oscillations. *Hydra*’s nerve nets are thus distributed, modular, and interact in dynamic patterns (Dupre and Yuste, 2017) (resembling graphs in Fig. 2D,E).

How do these ensembles synchronise their activity? *Hydra* possesses both chemical and electrical synapses, as described in classic ultrastructural studies (Westfall et al, 1971b, 1980). However, the recent vEM analysis of the endodermal nerve net found no synapses between any of the 20 interconnected neurons. There were also no synaptic connections between the endodermal and ectodermal nets (Zhang et al, 2025). In the synapse-free endodermal net, gap junctions at the identified neuronal handshakes may provide electrical coupling as the primary means of neuron–neuron communication. Single-cell transcriptomics revealed several innexin genes expressed in different neuron types (Keramidioti et al, 2024). The ectodermal nerve nets may synchronise their activity by synapses—present between ectodermal neurons (Westfall et al, 1971a)—or gap junctions, or both. Such dual signalling was described for the hydrozoan medusa *Aequorea* where swim pulses are generated by electrically coupled “swimming motor neurons” (SMNs) located in the inner nerve ring. These SMNs also receive small, graded synaptic inputs, indicating that electrical and chemical signalling act together to shape the dynamics of the swim motor network (Satterlie, 1985).

In ctenophores, the subepithelial (SNN) and aboral nerve nets (ANN) form multinucleate syncytial nets with a continuous cytoplasm (Burkhardt et al, 2023; Ferraioli et al, 2026; Jokura et al, 2025). It remains to be determined whether these syncytial nerve nets are non-polarised and through-conducting, as their anatomy suggests, or show regional activation with polarised spread. The aboral nerve net synapses on ciliated balancer cells and high-speed imaging of balancer cilia revealed coordinated regulation of beat frequency, arrests and re-beats (Jokura et al, 2025). This suggests that the ANN functions as a ciliary pacemaker and coordinator system that fine-tunes the balancers.

In the body wall of ctenophores, the syncytial net may mediate fast and spatially extensive conduction. While the aboral nerve net receives no synapses from sensory cells, sensory synapses were observed on the body-wall nerve-net (Burkhardt et al, 2023). Local input from such sensory cells may trigger directional spread of activity from the stimulated foci. Differences in SNN and ANN morphologies (‘beads on a string’ vs. uniform diameter) suggest different dynamics of activity spread, an exciting topic for future studies. Revealing the dynamics of these syncytial nets will require the establishment of calcium imaging for ctenophores.

## Signalling by nerve nets to effector organs

To coordinate actions, nerve nets eventually need to control effector activity. There are three ways to achieve this: direct synaptic innervation, electrical coupling via gap junctions, and volume transmission.

Ctenophore neurons form chemical synapses on many effector cell types, including ciliated cells, parietal muscles, mesogleal muscle fibres, colloblasts, and photocytes (Horridge and Mackay, 1964; Hernandez-Nicaise, 1973, 1976; Shelton, 1982; Franc, 1978; Anctil, 1985; Ferraioli et al, 2026; Jokura et al, 2025). Synaptic input can generate calcium- and sodium-dependent action potentials on the giant smooth muscle cells of *Beroë ovata* (Hernandez-Nicaise et al, 1980; Hernandez-Nicaise and Amsellem, 1980).

There is also evidence for direct electrical coupling from nerve net to effectors. Ultrastructural analyses have identified gap junctions between neurons and epitheliomuscular cells, as well as between neurons themselves (Westfall et al, 1980). This suggests that electrical coupling may contribute to the rapid coordination of muscle activity.

In *Aequorea*, swimming motor neurons (SMNs) located in the inner nerve ring are electrically coupled and function as pacemakers through intrinsic oscillations of their membrane potential. SMNs first depolarize a sheet of excitable but non-contractile epithelial cells. These epithelial cells are linked by gap junctions, and are in turn electrically coupled to the contractile, striated myoepithelial muscle sheet that surrounds the bell (Satterlie and Spencer, 1983; Satterlie, 2002b; Satterlie, 2008).

In a large number of cases, however, there are no direct synaptic connections between nerve net and effectors and signalling is likely mediated by diffusible transmitters by the process of volume transmission. During volume transmission neuropeptides or other transmitters (e.g. monoamines) are released along the neurites of source neurons and act on nearby receptor-expressing target cells.

The endodermal nerve net of *Hydra* provides a clear example. The recent vEM analysis of the endodermal nerve net not only failed to identify synapses between neurons, but also between neurons and effector cells. This is not due to low resolution or fixation artefacts, since in the dataset of the small *Hydra* that was reconstructed, all vesicles could be segmented and classified. This comprehensive analysis showed that vesicles are not clustered around potential target cells (Zhang et al, 2025). Consequently, the predominant mode of transmission from the nerve net to the epitheliomuscular cells is thought to rely on non-synaptic, neuroendocrine-like volume transmission. The diversity of vesicles suggests a mixture of small-molecule and neuropeptide transmitters, potentially acting on diverse targets. One candidate to elicit muscle contractions is the neuropeptide GLWamide. The GLWamide precursor is expressed in endodermal neurons and bath application of the peptide leads to the contraction of the endoderm and body-column elongation (Takahashi et al, 2003).

Physiological and behavioural studies demonstrate that several neuropeptides expressed in the ectodermal nerve nets are also major drivers of behaviours. Application of the peptide Hym-248 induces somersaulting behaviour (Yamamoto and Yuste, 2023). In this case, the peptide acts directly on the nerve-net, inducing an all-or-none ‘peptidergic action potential’ to drive a complex behavioural sequence. The nerve net likely signals to the muscles via synapses between the ectodermal net and muscles. Another peptide regulator or potential transmitter is the peptide Hym-176 that elicits strong contractions of the ectodermal muscle (Yum et al, 1998a, 1998b). An interesting distinction emerges between the peptidergic endoderm and the synaptic-peptidergic ectoderm in *Hydra*. Since the endoderm only drives slow radial contractions during feeding, chemical signalling may be sufficient for such slow behaviours. In contrast, rapid contractions elicited by the ectodermal nerve net require synaptic contact (Keramidioti et al, 2024).

In *Clytia*, the local folding of the bell margin during food handling is controlled by a subumbrellar network of RFamide-expressing neurons (Weissbourd et al, 2021). Transmission from this RFamide network to the smooth muscle sheet is thought to occur primarily through peptidergic volume transmission to regulate muscle excitability.

The systematic characterisation of a large number of neuropeptide receptors in *Nematostella vectensis* suggests extensive peptidergic signalling in this anthozoan and other cnidarians. Neuropeptides and their receptors are specifically expressed in different combinations of neurons. The potential ligand-receptor signalling connections form a complex chemical connectome. These chemical layers likely enrich the synaptic layer, potentially defining different states or driving behaviours (Thiel et al, 2024).

Bath application of neuropeptides in ctenophores have revealed additional cases of potential volume transmission. In *Bolinopsis mikado* cydippid larvae, NPWa induces contraction of the adradial canals and mouth opening, whereas VWYa triggers expansion of the oral epithelium and whole-body inflation (Hayakawa et al, 2022). Similarly, in *Mnemiopsis leidyi*, several neuropeptides expressed in the subepithelial nerve net (SNN) can alter larval behaviour when applied in bath preparations (Sachkova et al, 2021).

Among the bilaterians, further examples of volumetric nerve-net signalling are provided by hemichordates, acoelomorphs and echinoderms. In hemichordates and acoelomorphs, the nerve net is basiepithelial and is separated from the muscles by a basal lamina (Andrade López et al, 2023; Raikova et al, 2000a). Imaging by confocal microscopy did not find processes that cross the basal lamina and synapse on the muscles. This suggests that the transmitters and peptides released by the nerve net act via diffusion across the basal lamina. Similar volumetric signalling occurs in the sea urchin *Strongylocentrotus franciscanus*. In this case, no neuro-muscular synapses were detected by EM between the cholinergic plexus and the muscle fibres yet acetylcholine was shown to influence muscle contractility (Florey and Cahill, 1980).

## Flexibility of nerve nets

Nerve nets exhibit high flexibility and are capable of reorganizing during major structural changes such as growth, degrowth or regeneration, while maintaining function.

Romanes was the first to describe the extensive structural resilience of nerve nets through his incision experiments in jellyfish (Romanes, 1885). He noted that “The extent to which the neuro-muscular tissues of the Medusa may be mutilated without undergoing destruction of their physiological continuity is in the highest degree astounding”. (quoted by Satterlie (2002b)) He also observed the “astonishing rapidity with which the excitable tissues of the Medusae regenerate themselves after injury.”

This flexibility is due to the distributed nature of nerve nets, their ability to add new neurons to an existing structure, to regenerate completely, and even fuse together in chimeric individuals.

In *Hydra*, new neurons are incorporated laterally into existing bundles during growth, and during regeneration the entire nerve net is rebuilt within the newly established body axis. Despite this continuous structural turnover, the timing of behaviours such as contraction, elongation, bending, and somersaulting remains unchanged. This indicates that function is stably maintained even as the architecture is remodelled (Keramidioti et al, 2024). *Hydra* also possesses independent oral and peduncle networks, and spontaneous and mechanically induced contractions recruit different subnetworks. Even when the peduncle network is removed, mechanosensory responses reappear during regeneration (Badhiwala et al, 2021). These findings show that *Hydra*’s nerve net is locally modular and resilient to damage.

In *Nematostella*, the body length expands and contracts depending on feeding state, and neuron numbers in both the ectodermal and endodermal nets change reversibly in parallel. When the animal is bisected, the regenerating polyp reconstructs a nerve net with fewer neurons, yet the network scales appropriately to the reduced body size and fully restores behaviours such as feeding, contraction, and touch responses (Havrilak et al, 2017). The nervous system also regenerates after an almost complete non-surgical, chemogenetic ablation (Mazloumi Gavgani et al, 2025). Amputation experiments with such nerve-free polyps also revealed an unexpected function of the nervous system in defining axial polarity during whole-body regeneration.

In ctenophores, flexibility takes an even more striking form. Two injured individuals are able to fuse to form a new integrated chimeric organism. The originally independent nervous systems of the two halves become fully synchronized, with left and right lobes adopting identical contraction rhythms (Jokura et al, 2024). This rapid integration likely does not require construction of new neural circuits. Instead, it suggests that the syncytial nerve net is able to establish coupling possibly by fusion, allowing coordinated motor output across the chimeric individual.

Nerve nets can also mediate flexible behaviours and are modulated by internal state. Sleep modulates the rhythmicity of bell contractions in *Cassiopea* medusae (Nath et al, 2017). In *Clytia*, the folding of the umbrella margin is modulated by hunger and reproductive state. When food is captured by multiple tentacles, food transfer to the mouth is sequential, indicating that beyond regional autonomy, the different sectors of the umbrella are globally coordinated (Weissbourd et al, 2021).

## Evolution of nerve nets

**Figure 5:**
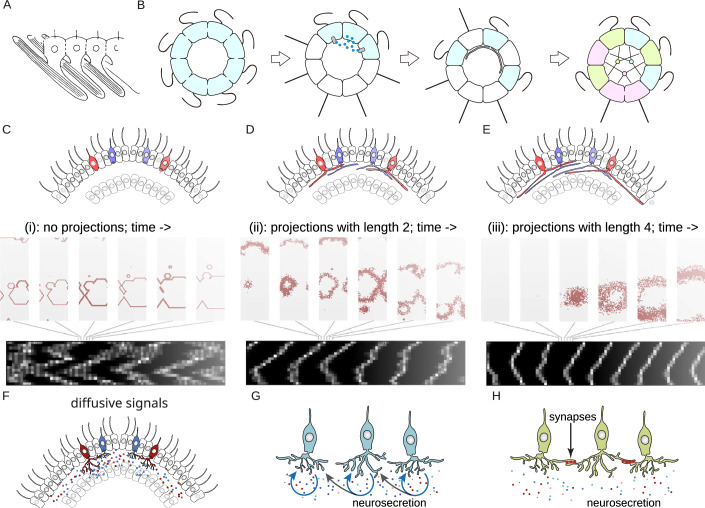
Evolution of nerve nets—‘wiring first’ and ‘chemical first’. (**A**) Schematic by Mackie of a proto-neuron where excitability and contractility coexist. (**B**) Small colonies of ciliated cells that are electrically coupled; here, the effector system is simplified to cilia alone. Electrical signals spread between cells, and the ciliary motor output of the coupled cells follows the same spatiotemporal pattern. Later, diffusible signals and projections appear and a nerve net evolves. (**C**–**E**) Modelling the spread of activity waves on an epithelial surface coordinated by excitable cells with projections with increasing length. After de Wiljes et al (2017). (**F**) Sensory-neurosecretory neuron with branched projections filled with dense-core vesicles for neuropeptide release. Tiling an epithelium with cells of the same type releasing the same peptide under the same conditions allows tissue-wide signalling. (**G**) Schematic of autocrine amplification of neurosecretion. (**H**) Schematic of synaptic coordination and amplification of neurosecretion. Autocrine signalling can allow spread and amplification.

The protistan ancestors of animals were single cells or small colonies, possibly akin to modern choanoflagellates (Cavalier-Smith, 2017). In these ancestors, the sensory, control and effector layers were combined in a single cell or a colony of cells. With the advent of Metazoa, multicellularity created a problem of coordination and control (Arnellos and Keijzer, 2019) (Fig. 5). Nerve nets may have provided the earliest solution to the coordination and control problem of early animals (Keijzer et al, 2013; de Wiljes et al, 2017).

Several accounts of nervous-system origins discuss the idea of nerve nets as coordinators of tissue-scale activity (Kleinenberg, 1872; Pantin, 1956; Mackie, 1970). In Mackie’s ‘neuroid conduction’ model, conduction and contraction co-occurred in a single planar tissue of myoepithelial cells connected by cytoplasmic bridges. Later, contractile cells segregated from neuronal cells, the neuronal cells lost their contractile component but coordinated the activity of the evolving muscles (Mackie, 1970).

Keijzer’s “skin–brain” thesis also proposes that early nervous systems evolved to regulate body-wide contractions (Keijzer et al, 2013). Most theories focused on contractility alone but were later extended to also emphasise the importance of ciliary control (Jékely, 2010), glands (Jékely et al, 2015) and the need for parallel solutions for ciliary and contractile systems (Arendt, 2021).

In contrast to these ‘wiring first’ theories, ‘chemical first’ theories emphasize diffusible signals as the earliest evolutionary solution for tissue-wide control (Grundfest, 1964; Lentz, 1968; Jékely, 2021; Moroz et al, 2021) (Fig. 5). In these theories, neurons evolved from ancestral secretory cells that may also have been sensory. These cells released chemicals that acted by volumetric signalling and formed complex chemical networks to integrate processes across tissues. Such signals could have been mediated by neuropeptide-like molecules, biogenic amines, other small transmitters or gaseous molecules such as nitric oxide. If chemical signals indeed originated before classical neurons with projections and synapses, then the emergence of neurons and nerve nets has to be discussed in the context of pre-existing chemical networks.

Why did the first nerve nets evolve? We offer possible explanations both in light of the ‘wiring first’ and the ‘chemical first’ theories. We discuss three steps, including the segregation of the effector layer from the sensory-control layer, the origin of projections and the origin of conducting nerve nets.

First, we look at these steps from the perspective of the ‘wiring first’ theories (Fig. 5). Mackie addressed the question of what could have driven the segregation of the effector layer from the sensory-control layer. He speculated that pacemaker activity could have been an early function for nerves, since the epitheliomuscular system in medusae lacks pacemaker capability (Mackie, 1970). Mackie also argued that nervous systems could have allowed the local excitation of effectors, increased conduction speed, and improved sensory processing and complex motor control due to insulation and specificity (Mackie, 1970).

Projections may have evolved to enhance the spread of activity. As shown by simple but elegant evolutionary simulations, even short, random projections could enhance the spread of contractile waves on larger surfaces (de Wiljes et al, 2017) (Fig. 5C–E). At which point pacemakers came into play is an important question to consider. Further studies on rhythmic behaviours (e.g. contractions, feeding cycles, oscillatory dynamics) of synapse-less sponges and placozoans could give important information (Nickel, 2004; Ueda et al, 1999; Nikitin et al, 2023).

In ‘chemical first’ scenarios, the growth of projections could have concentrated secretory activity at the end of the extensions (Grundfest, 1964) (Fig. 5F–H). Projections from uniformly spaced neurons could also have increase the total membrane surface for transmitter release (Jékely, 2021). These projections eventually enabled cells in the nerve net to connect to each other (either as syncytia or via electrical or chemical synapses) to facilitate the spread of activity within a nerve net (Fig. 5F–H).

In these theories, the earliest nervous systems appear less as simple sensory-motor reflex arcs (input-output models)(Jékely et al, 2015) and more as electrically coupled sheets, peptide-secreting cells or emerging neurons forming nets and sharing a common conductive field. Another interesting question is when neuronal polarity evolved and why (Rolls and Jegla, 2015).

Chemical-first and wiring-first scenarios are not necessarily mutually exclusive. A plausible intermediate regime involves a division of labour in which (i) diffusible signals such as peptides and monoamines (Yañez-Guerra et al, 2026) tune global excitability (i.e., organism-wide state), while (ii) short-range coupling via synapses supports local propagation.

## Origins of nervous centralisation

In contrast to nerve nets, in centralised nervous systems neuronal somata cluster together into ganglia or brains and the neurites form chords or bundles (Arendt, 2018a). The origins of nervous centralisation has been extensively debated (Holland, 2003; Northcutt, 2012; Stach et al, 2012; Denes et al, 2007; Martín-Durán et al, 2017; Arendt, 2018a). Most of the discussion has focused on the origin of an anterior brain and ventral nerve cords, as commonly seen in bilaterians. While centralised nervous systems are common, including in vertebrates, annelids or arthropods, many bilaterians show much less centralisation, including hemichordates, acoels or echinoderms. A central question thus has been the presence or absence of centralisation in the urbilaterian (last common ancestor of bilateria). The analyses addressing this question have often been coarse-grained and simply assigned either of two character states (nerve net, vs. centralised nervous system) to entire animal phyla (Northcutt, 2012).

Before turning to the urbilaterian case, we would like to highlight some examples for the independent evolution of condensations or decondensations. As we have seen above, all animals with a diffuse or nerve-net-like nervous system deviate from a random null pattern. In addition, all animals with a nerve net also show signs of centralisation to varying degrees.

Even in animals like *Hydra*, there are nervous condensations around the foot and the mouth, including a hypostome nerve ring (Grimmelikhuijzen, 1985; Koizumi et al, 1992; Takahashi et al, 2003; Koizumi, 2007). Anthozoans likewise have nervous centres. In *Nematostella*, there is a centralised oral nerve ring showing ganglion-like condensations (Zhong et al, 2026).

A high degree of centralisation is found in the sensory rhopalia of cubozoan jellyfish (Nielsen et al, 2021). In these animals, the four rhopalia serve as swim pacemakers and visual centres. Each rhopalium contains two lensed camera eyes, four ocelli, a statolith and a complex neuropil with various nerve tracts that connect the sensory structures. A complex ring nerve connects the four rhopalia across the animal, enabling global integration (Satterlie, 2011). The highly diffuse and non-polarised nerve net in scyphomedusae also connects to centralised rhopalia. There are eight or more rhopalia in a scyphomedusa that form ganglionic sensory (with light- and mechanosensory activity), integrative and pacemaker centres (Satterlie, 2011).

Among the Bilateria, nerve nets often co-occur with more centralised nerve elements and can show various levels of centralisation within a phylum. In Xenacoelomorpha, the nervous system likely underwent multiple, independent episodes of neurite-bundle condensation (Gavilán et al, 2016; Achatz and Martinez, 2012) and decondensation. Acoels, nemertodermatids, and *Xenoturbella* show many different combinations of diffuse basiepidermal nets and longitudinal cords that evolved independent from an ancestral bilaterian CNS plan (Martín-Durán et al, 2017). The divergent molecular profile of dorsoventral patterning across Xenacoelomorpha suggests that condensations arose by lineage-specific reorganisations of peripheral nerve nets rather than through modification of a pre-existing central cord. To the contrary, some groups within Xenacoelomorpha evolved a more diffuse organisation, including *Hofstenia* (Hulett et al, 2020; Martinez et al, 2024) or the parasitic nemertodermatid *Meara stichopi* (Raikova et al, 2000b).

Centralisation can thus come and go in evolution, and we do not have a good understanding of how function and behaviour change with it. Such large anatomical reorganisations are unlikely to occur through neutral evolutionary change, given that nervous systems are expensive and wiring-optimised (Chen et al, 2006). Neural function, anatomy, behaviour and habitat are thus important to consider when discussing the evolution of centralisation.

One example is provided by organisms covered with motile cilia used to move between sand grains or glide on surfaces. In the Acoelomorpha, changes in nervous system architecture were linked to adaptations to an interstitial lifestyle. These include a multiciliary epidermis that may have derived from a ciliated larval stage by progenesis (Achatz and Martinez, 2012). We hypothesize that the peripheral nerve nets observed in acoels (and also in hemichordates) are diffuse because they need to innervate ciliated cells across the entire surface of the body to control them. In animals with ciliary gliding, such as nemerteans or flatworms, cilia can be concentrated ventrally in a mucociliary sole and receive more centralised innervation (Arendt et al, 2015). In these cases, ciliary control, habitat and nervous system anatomy may be intimately linked in ways we do not yet understand.

In *Saccoglossus kowalevskii*, the nervous system is dominated by a basiepithelial plexus, with only modest dorsal and ventral cords and no anatomically discrete CNS (Andrade López et al, 2023). The nervous system is regionally patterned along the anterior-posterior axis, but there are no medio-lateral territories as in bilaterians with centralised nerve cords but ring-like gene-expression territories (Lowe et al, 2003; Denes et al, 2007). Whether this non-centralised architecture is due to evolutionary loss remains contested.

Conservation in gene-expression programmes that specify neural cell types along different mediolateral territories in the annelid trunk was taken as evidence that a centralized nervous system existed in the urbilaterian (Denes et al, 2007). In *Platynereis*, the Nk2–Pax6–Dlx arrangement closely mirrors that of vertebrates, suggesting deep homology (Denes et al, 2007). However, broader comparisons show that this organisation is not universal (Martín-Durán et al, 2017). One reading of these data is that centralisation in bilaterians reflects repeated condensation of peripheral nerve nets using a shared developmental toolkit rather than the elaboration of a single ancestral CNS (Martín-Durán et al, 2017).

A counter-argument is that no positive characteristic unites all diffuse bilaterian and other nervous systems as a plesiomorphic trait. As we discussed, the nerve nets in scyphozoans, hemichordates, nemertodermatids, ctenophore and other groups can show fundamentally different fine-scale cellular organisations. Simply put, being ‘diffuse’ or ‘net-like’ is not a useful characteristic. In contrast, many centralised nervous systems across Bilateria show molecular similarities in the mediolateral patterning of their constituent cell types (Arendt, 2018a). To paraphrase Tolstoy, all centralised nervous systems are alike; each non-centralised nervous system is non-centralised in its own way.

In light of the above, one possible scenario is that centralisation in cnidarians and bilaterians has common origin, as proposed by Francis Balfour (Balfour, 1880) and later by e.g. Osamu Koizumi (Koizumi, 2007). In Balfour’s view, the ancestral nervous system was ‘a circumoral ring, like that of Medusae, with which radially-arranged sense-organs may have been connected.’ This then evolved into the bilaterian nervous system: ‘a circumoral nerve-ring, if longitudinally extended, might give rise to a pair of nerve-cords united in front and behind’.

A condensed nerve ring, potentially with ganglionic concentrations as in *Nematostella* (Zhong et al, 2026) may represent the ancestral condition in Cnidaria. The outer nerve ring in many cnidarians integrates sensory input (Spencer and Arkett, 1984). Sensory cells run radially and connect to bipolar ganglion cells that form the nerve ring (Koizumi, 2007). A segmental ganglionic nervous system as seen in many bilaterian could have evolved by converting the radial body segments (mesenteries in anthozoans) to longitudinal segment (Arendt, 2018b; He et al, 2018). The vEM-enabled discovery of a ‘mechanosensory girdle’ in the annelid larva with sensory cells projecting medially and synapsing on bipolar interneurons supports this view (Verasztó et al, 2025).

## Conclusion and future directions

Notions about all nerve nets representing an ancestral condition are widespread. These are in part perpetuated by ladder-thinking (i.e. reading phylogenies as ladders of progress (Omland et al, 2008) instead of branching trees) that is pervasive in the literature on cnidarians. Statements like Cnidaria represent “the first group of animals to evolve a recognizable nervous system” are obviously wrong since nervous systems evolved in the stem lineage leading to Bilateria and Cnidaria. We also have no way of telling if this was before or after ctenophores evolved a nervous system (if they did so independently (Moroz, 2009, 2015)).

The most advanced nerve nets in scyphozoan medusae are not preludes to something better. They are highly evolved solutions for coordinating a muscular umbrella for water-jet propulsion in these successful pelagic predators. It may indeed be coming of age of scyphozoans and their advanced nerve nets while maladaptive fish is caught by other types of nets en masse by an apex predator (Calbet, 2025).

Nerve nets in combination with distributed sensory-integrative-pacemaker centres, such as in jellyfish, represent a fascinating organisational principle. This contrasts with a centralised single-command-centre architecture and may be more resilient to damage, hijacking or even disease. The distributed organisation of nerve nets could inspire improvements in diverse man-made systems, including computing, transportation, administration, or social media (the Fediverse vs. a single centralised platform). If we stopped considering animals with nerve nets as ‘primitive’ we could start learning from them.

## Supplementary information

Peer Review File
