# Supplementary material for: Revisiting nerve nets: functional organization and evolutionary implications
Source: EMBO J. 2026 Jul 23;45(16):5607–21. doi: 10.1038/s44318-026-00879-w (PMC13482580; doi:10.1038/s44318-026-00879-w)
Supplement: Supplementary file 1 [file 44318_2026_879_MOESM1_ESM.pdf]

THE EMBO JOURNAL

# Revisiting Nerve Nets: Functional Organization and Evolutionary Implication

Kei Jokura and Gáspár Jékely

*Corresponding author(s): Gáspár Jékely (gaspar.jekely@cos.uni-heidelberg.de)*



## Review Timeline:

| | |
|---|---|
| Submission Date: | 17th Dec 25 |
| Editorial Decision: | 3rd Feb 26 |
| Revision Received: | 14th Feb 26 |
| Editorial Decision: | 2nd Mar 26 |
| Revision Received: | 23rd Jun 26 |
| Editorial Decision: | 7th Jul 26 |
| Revision Received: | 9th Jul 26 |
| Accepted: | 13th Jul 26 |

*Editor: Yehu Moran*

## Transaction Report:

(Note: With the exception of the correction of typographical or spelling errors that could be a source of ambiguity, letters and reports are not edited. Depending on transfer agreements, referee reports obtained elsewhere may or may not be included in this compilation. Referee reports are anonymous unless the Referee chooses to sign their reports.)

## 1st Editorial Decision 3rd Feb 2026

Dear Prof. Jékely,

Thank you for submitting your manuscript to The EMBO Journal. It has now been evaluated by two referees, whose comments are enclosed. A third reviewer who had agreed to review has unfortunately not yet delivered their report, and we will therefore proceed based on the available evaluations. I sincerely apologize for the delay created by waiting on the third reviewer.

Given the strong interest expressed by both the referees and the editorial board, we would in principle be pleased to consider a revised version of your review for publication. However, as you will see, the referees raise a substantial number of comments that will require careful attention.

The hard deadline for inclusion in the special issue on Ecology and Evolution is 13 February. It seems unlikely that a thorough revision could be completed within this timeframe. However, alongside the special issue we are launching a continuously curated Ecology and Evolution series at The EMBO Journal, and your manuscript can certainly be included in that series once accepted.

Please let me know how you wish to proceed.

When preparing your letter of response to the referees' comments, please bear in mind that this will form part of the Review Process File, and will therefore be available online to the community. For more details on our Transparent Editorial Process, please review our Editorial Policies page: https://link.springer.com/partners/embo-press/editorial-policies

We generally allow three months as standard revision time. Yet, in light of the special issue and special series we will appreciate it very much if you could submbit your revision sooner.

Thank you for the opportunity to consider your work for publication. I look forward to your revision.

Yours sincerely,

Yehu Moran
Academic Editor
The EMBO Journal

Instructions for preparing your revised manuscript:

Read our guidance for manuscript revisions and related editorial policies: https://link.springer.com/journal/44318/submission-guidelines#cms-Revised-submissions

Please also check that the title and abstract of the manuscript are brief, yet explicit, even to non-specialists.

When assembling figures, please refer to our figure preparation guideline in order to ensure proper formatting and readability in print as well as on screen:
https://media.springernature.com/original/springer-cms/rest/v1/content/27825798/data/v1

IMPORTANT: When you send the revision we will require
- a point-by-point response to the referees' comments, with a detailed description of the changes made (as a word file).
- a word file of the manuscript text.
- individual production quality figure files (one file per figure)
- a complete author checklist
- Expanded View files (replacing Supplementary Information)
- a Reagents and Tools Table as part of the Methods section

Please remember: Digital image enhancement is acceptable practice, as long as it accurately represents the original data and conforms to community standards. If a figure has been subjected to significant electronic manipulation, this must be noted in the figure legend or in the 'Methods' section. The editors reserve the right to request original versions of figures and the original images that were used to assemble the figure.

We realize that it is difficult to revise to a specific deadline. In the interest of protecting the conceptual advance provided by the work, we recommend a revision within 3 months (4th May 2026). Please discuss the revision progress ahead of this time with

the editor if you require more time to complete the revisions. Use the link below to submit your revision:

https://emboj.msubmit.net/cgi-bin/main.plex

------------------------------------------------

Referee #1:

Review summary

This review aims to challenge a view of nerve nets as primitive, random associations of neurons, arguing instead that they represent sophisticated and enduring systems optimized for distributed control. By synthesising evidence from classical studies with modern data from volume electron microscopy and transgenenics, the authors illustrate how nerve nets in cnidarians, ctenophores, and other lineages possess a high degree of structural and functional complexity that defies a "null hypothesis" of a disorderly network. They cover a wealth of literature showing how these systems exhibit precise developmental patterning, distinct neuronal cell types, stereotyped neurite projections, and specific, often polarized, synaptic connectivity.

The review highlights the diversity of neuroanatomy in nerve nets, e.g. connectomic reconstruction of the Hydra endodermal net that reveals a synapse-free architecture that likely uses electrical coupling, while ctenophores like Mnemiopsis feature syncytial nerve nets that function in a continuum to coordinate ciliary beating.

The paper highlights function and physiology as well as anatomy, e.g. how nerve nets achieve organism-wide coordination through diverse mechanisms, including through-conduction, regionally autonomous ensembles, and volume transmission. They discuss how in jellyfish, specific sub-nets drive behaviours like umbrella infolding, and how computational models show that simple local interaction rules can generate complex, global activity patterns without the need for centralized command hubs. The authors highlight the ctenophore aboral nerve net as a coordination network that synchronizes balancer cilia, via specific synaptic targets.

These examples and themes are put together to illustrate that that the diffuse architecture of nerve nets is not merely an evolutionary stepping stone or a primitive remnant, but a convergent, highly evolved solution ideal for the locomotion and signalling requirements of radially symmetrical, soft-bodied animals.

Overall, the manuscript is free of major errors or inaccuracies and covers a lot of ground. Many of the figures are of high quality and informative. The literature is extensive but could benefit from a few additions (detail below). The main downside is that the writing is rather exhausting and disconnected. I sympathise with the challenge the authors have in synthesizing a large, disparate volume of information into a coherent article, but I think they could do a better job if they lead each section with more compelling questions that they then answer, using the literature and examples.

With careful restructuring and fixes to some of the writing (detail below) the article will make a useful addition, drawing together literature that is often overlooked in a prominent venue. But it needs to draw readers in and convince outsiders to this topic that there are compelling, big picture lessons that they can take away.

Main comments

The opening section "what is a nerve net" lays out the purpose of the article but does so vaguely. It would help to lay out the claims and contributions explicitly: for example, are we questioning the definition of a nerve net? Are we making claims about evolutionary origins or function? A pointed paragraph will let the reader know what to expect and allow the reader to evaluate whether the article delivers its promises.

In the next section the authors state that "A null hypothesis for nerve nets would posit a loose and disorderly organisation" why? What would this imply and why would deviation from it be informative? Has the claim they are random been made, and if so by whom? One could argue that simpler nervous systems would have more stereotypical organisation, with the wiring more constrained by a simple and rigid developmental program, for instance. The hypothesis seems to be a straw man, in other words, and its main purpose seems to be to point out that structure varies (but so what?). This should be motivated by more convincing and granular hypotheses. Doing so would give better structure and interpretability to the numerous paragraphs on pages 4-5, which currently reads as a rather exhausting list of facts.

The section on connectomics should cite more of the mainstream connectomics literature and methodologies (e.g. in C elegans, Drosophila, mouse....). It would be helpful to describe some of the challenges and limitations of the methodology, especially in applying it to new species. This section does not bring home any clear points, other than mentioning the existence of a connectome (from single/multiple specimens?) with a throwaway sentence at the end that doesn't provide an insightful conclusion. The reader is left wondering what we learned from connectomics in these species and what we stand to learn.

The section on Spread of activity (P7 onwards) would really benefit from diagrams showing modes of how activity propagates in the different systems mentioned.

The conclusion could do with something bolder. "Future directions" being to study nerve nets in more detail is not very compelling. The authors should try to consider what non-specialist readers should take away from the article and how the study of nerve nets changes our thinking about neurophysiology, development, evolution and so on.

Minor comments/typos/grammar/clarification

Figure 1: would benefit from a (abbreviated!) phylogenetic tree to illustrate how diverged the species are.
P1 "The other is theories" - pluralisation?
P1 "sometimes lump vastly different diffuse organisations as one character state" - clarify
P3 "endodermal nets are very differently organised" - vague, clarify
P3 "neuronal markers revealed a layered organisation" - tense
Figure 3: panels are too close together and text is too small in panels D and E
P8 "Electrical recordings from hydromedusae found that their nerve nets show mor regional autonomy than in scyphomedusae" - orphaned sentence, should be in a paragraph
P10 "The nerve net likely signals to the muscles by synapses, present between..." clarity - consider rewriting to "The nerve net likely signals to the muscles via synapses between..."
P16 (and throughout) "Simply put, being 'diffuse' is not a useful character" - I think you mean "characteristic". Consider substituting throughout the article as this confused me.
P16 "perpetuated by ladder-thinking (instead of tree-thinking)" - I understand what you mean but some readers may benefit from a follow on sentence expanding on this metaphor.

Referee #2:

The author's manuscript is a timely and exciting topic that discusses classical and recent findings about nerve nets. The review covers anatomical, developmental, physiological and evolutionary aspects over a range of different phyla that possess this type of nervous system. The authors conclude that nerve nets are diverse, often show a polarized organization and functional specialization. Nerve nets vary not only in anatomy but also in transmission architecture, using chemical synapses, gap junctions or syncytial fusions as well as volume transmission through neuropeptides as means of connecting neurons with each other and to sensor and effector cells. These findings put together, and in perspective, hint towards an extremely intricate and complex system of behavioral control and signal processing through nerve nets.
While the topic and the papers covered here are certainly exciting and the review will be interesting for a broad readership, the manuscript is not well structured. Unfortunately, concepts and lines of thought, are mixed and make it very difficult to follow the ideas and text flow, specifically, if one is not an expert in the field. Animals from different phyla and subphyla are mixed and phylogenetic relationships are not considered with respect to the nerve net organization. Hypotheses are not presented logically and may even be opposed a few paragraphs later which is confusing. While I feel that this review will be beneficial to the field, I strongly recommend a rigorous editing
Below please find some more detailed examples:

Major concerns:

• The manuscript is not well structured, concepts and lines of thought, are mixed and make it very difficult to follow the ideas and text flow, specifically, if one is not an expert in the field.
• The text is too detailed for a review article, especially in some aspects (e.g. Ctenophore nerve net) and many passages read like summaries of original research rather than a review. Concretely, this means that non experts may have to read primary literature to understand large parts of the text. This is also prevalent later in the text in which the mathematical modeling part reads like a summary of the paper.
• Animals from different phyla and subphyla are mixed and phylogenetic relationships are not considered with respect to the nerve net organization and other speculations. Generalizations might therefore better be used with care.
• Some hypotheses (especially in the first part of the manuscript) are not presented logically and may even be opposed a few paragraphs later which is confusing. The concepts underlying the formulation of the hypothesis need rigorous work. This is especially important as there are not just nerve nets and brains but a variety of intermediate neuronal structures (which the authors discuss at the very end- it might be beneficial to consider restructuring the organization of the text).
• Definitions, Figures and Schemes are not presented logically, come several paragraphs later or are non-existent. For example diffuse, disorganized, random, disorderly and unpolarized are used without definition and interchangeably. The current text without clear definitions can be highly confusing and would benefit from an initial explanation (definition) of the different terminology, followed by a consistent use of those words in their respective context.
• Many Figures are not mentioned at the respective text passages or at all (for some aspects of the figures (e.g. subfigures A,B...), while difficult concepts are not depicted in the figures.

• The authors speculate on many aspects and often the speculations are not connected by structured analysis transferring observations and concepts between phyla, subphyla or species. This is particularly evident with respect to inference of physiological functions without solid molecular and cellular physiology present in many of the described species. The authors infer function across different species, from transcriptomics or anatomy. In my opinion this is not best practice, and such formulations should be labelled as theories or speculations that await further analysis.
• English grammar, text composition, words and tense are incorrect throughout the text.
• Many passages cannot be understood without being familiar with the primary literature and are very detailed and complicated while elsewhere critical information is missing.
• I am not familiar with all literature, but some content is reflected incorrectly (see examples below) and these points are not reflected critically or appear to be selectively used to support arguments of the authors.
• I highly recommend shortening the manuscript. Many of the later theories are discussed extensively elsewhere.
Specific examples:
-first paragraph: both species and definition seem confusing: many animals within the list possess ganglia like structures, such as Rhopalia, apical organs, nerve rings and neuronal concentrations e.g. hydra and anemones around foot and mouth region etc in which concentrations and polarity are found. Seems good to reconsider phrasing here. In addition, the authors make the very same point a few paragraphs later and discuss these points towards the end of the text.

- "A null hypothesis for nerve nets would posit a loose and disorderly organisation." Do not understand this sentence, also as mentioned before: just by the fact that these animals possess structural and tissue organization, sensor and effector cells/tissues there is an inherent polarization to the nerve net- the authors must formulate their thoughts and the origin of this hypothesis more clearly. A few paragraphs later they state: "Nerve nets of a fully random organisation likely do not exist", thereby nullifying a theory whose origin was not clear to begin with.

-" In hemichordates - bilaterian animals with a diffuse nerve plexus and belonging to the ambulacraria": as mentioned previously- it would be good if there were some definitions, specifically in animals where neuronal structures are forming intermediate structures between NN and centralized aspects. Nerve plexus here means nerve net?

-" Second, neurons within nerve nets show functional specialisations.": This is highly speculative as different transcripts and morphology must not necessarily equal different functionality. At the very minimum, the authors should support this claim with a functional study or reformulate this sentence.

-"In the ctenophore subepithelial nerve net (SNN), no anatomically polarity has been observed.": this paragraph is in the middle of neurite polarization. Might be better to reorganize this paragraph?

-"However, this nerve net is also well organised with uniform tiling of the body.": To me, it is unclear what this sentence means.

-Under cellular organization, the authors state that the synapses are non-polarised and bidirectional. A few paragraphs later, the authors reverse this statement: "Fourth, synaptic connectivity in nerve nets is also non-random and follows clear structural rules andsynaptic polarity (with defined pre- and postsynaptic partners)."

-" tip-to-tip "neuronal handshakes"" I recommend to change this formulation for clarity, how is this not random? And what would be random? The definition comes several lines later: In Hydra, the endodermal nerve net of a small animal has been reconstructed. "This nerve net is formed of 20 neurons wherein neurons only connect to their immediate neighbours. Surprisingly, no classical chemical synapses could be identified in this dataset. Instead, neurites of adjacent neurons formed digiform tips, which interdigitated with partner neurites. These structures - termed neural handshakes - were devoid of vesicles indicating that they do not function as classical synapses. It remains to be determined if these handshakes contain gap junctions, as seems likely (Keramidioti et al, 2024a)."

-" The bidirectional synapses observed in cnidarians (Anderson & Schwab, 1981) and also ctenophores (Westfall, 1996) constitute a special case that likely occurs where non-directional transmission is an advantage, such as within the swimming-motor nerve net of jellyfish. Such nonpolarised transmission may not represent the default or ancestral state." This statement is highly speculative, and the point is reversed as mentioned elsewhere

-" In a pioneering study, Peteya was able to infer the overall synaptic connectivity of the ectodermal nervenet of the anthozoan cerianthid Ceriantheopsis americanus Peteya (1973)." This is not vEM- the text flow here as also in many other places is extremely misleading

"Instead, neurites of adjacent neurons formed digiform tips, which interdigitated with partner neurites." What does this mean? "Digiform" is not an English word- if anatomical description, this needs to be explained.

-the description of the ctenophore ANN is very difficult to comprehend

-" This spread is nonpolarised as first shown by Romanes in a series of elegant incision experiments (Romanes, 1885) (Figure 2). When he incised the umbrella in complex patterns, he found that the transmission of the excitation could pass around the incisions, showing diffuse and nonpolarised spread." This statement should be toned down as later Mackie, Passano and others showed that hydroids have neuroid conduction via epithelia which can explain the results of the incisions, see also: "Neuroid conduction and the evolution of conducting tissues" by G.O. Mackie 1970

-"In the body wall, the singular and extensive syncytial network may mediate fast and spatially extensive conduction." This is highly speculative and very unclear to me how this would work physiologically.

-"These likely form diffusion barriers and a slow the spread of activity, potentially limiting activation to local sectors." This statement is speculative and potentially wrong- nodes of Ranvier are also constricted and potentiate spread of activity. As mentioned elsewhere, it would be desirable not to infer physiological function from anatomy.

-"One classical example is provided by Hernandez-Nicaise et al. (1980), who showed that the giant smooth musclecells of Beroë ovata receive dense chemical synaptic input from the nerve net and are capable of generating sodium-dependent action potentials (Hernandez-Nicaise & Amsellem, 1980)." This Paper actually states that the AP is carried by both sodium and calcium, see also Liebeskind et al 2011- Ctenophores most likely possess only calcium selective Nav channels.

-"In Nematostella, body length expands and contracts depending on feeding state, and neuron numbers in both the ectodermal and endodermal nets change reversibly in parallel. When the animal is bisected, the regenerating polyp reconstructs a nerve net with fewer neurons, yet the network scales appropriately to the reduced body size and fully restores behaviors such as feeding, contraction, and touch responses (Havrilak et al, 2017)." Here newer literature might be incorporated: Gavgani et al 2025 also show that the nervous system is relevant to guide recovery.

-"Instead, it reflects the ability of the syncytial nerve net to establish electrophysiological coupling and a newly continuous tissue, allowing coordinated motor output across the chimeric individual." In my opinion, electrophysiological is both ambivalent and speculative here: this could rely on other mechanisms such as volume transmission, however, physiological proof is required to make any such statement.

-"These ancestors were isopotential and the sensory, control and effector layers were combined in a single cell or an electrically coupled colony of cells. The" Not all protists are isopotential, see for example G.O Mackies 1970s Neuroid conduction and the evolution of conducting tissues: Noctiluca. As mentioned elsewhere, best not to generalize these statements, there may be much out there that we do not yet know or understand. It is also unlikely that the fused choanoflagellates mentioned below are isopotential.

-" In general, a pacemaker circuit would require some feedback and multi-neuron ensemble, which suggests that projections and synapses came before pacemaker activity." It is unclear to me how the authors come to this conclusion- pacemakers can be found on a molecular level too.

Figures:

Fig1:
-Figure and text are not in harmony: Here organized and polarized NN are shown, with concentrations of neurons into sensory organs, while the authors discuss the opposite in the text

Fig2: In connection with Figure 1 and logic error in the text: It is very unclear where the hypothesis (or facts) of a disorganized nerve net come from. This theory would gain strength if the authors had some examples of where or why they assume that the net is "random and unpolarized", Figure 2 is only mentioned once in the text. 2E) looks similar to Weissbourd et al 2021 - may be appropriate to cite the paper here.

Figure3: Is not connected to the text, specifically for the explanation of the ctenophore NN this will be very helpful

Figure 4 and 5: subparts are not mentioned or connected to the text

Minor aspects:

-check citations, some are annotated wrong in the text e.g. (Havrilak et al, 2025) is actually 2017
-abbreviations are not introduced (e.g. AP anterior posterior)
-speculations about function and physiology must be clearly labelled as such
-best not to use generalizations like "in all other nerve nets" given the small number of organisms in which those have been sampled and analyzed
-spreading of electrical activity, electrical coupling: speculative

-Volume transmission: introduce concept
-the five aspects the authors are describing could be perceived more structured with subheadings
-development- more and newer citations for cnidarian nerve centralization
-"The aboral nerve net synapses on ciliated balancer cells and highspeed imaging of balancer cilia revealed coordinated regulation of beat frequency, arrests and re-beats." Citation missing

# Response to reviewers

## Referee #1:

### Review summary

This review aims to challenge a view of nerve nets as primitive, random associations of neurons, arguing instead that they represent sophisticated and enduring systems optimized for distributed control. By synthesising evidence from classical studies with modern data from volume electron microscopy and transgenenics, the authors illustrate how nerve nets in cnidarians, ctenophores, and other lineages possess a high degree of structural and functional complexity that defies a “null hypothesis” of a disorderly network. They cover a wealth of literature showing how these systems exhibit precise developmental patterning, distinct neuronal cell types, stereotyped neurite projections, and specific, often polarized, synaptic connectivity.

The review highlights the diversity of neuroanatomy in nerve nets, e.g. connectomic reconstruction of the Hydra endodermal net that reveals a synapse-free architecture that likely uses electrical coupling, while ctenophores like Mnemiopsis feature syncytial nerve nets that function in a continuum to coordinate ciliary beating.

The paper highlights function and physiology as well as anatomy, e.g. how nerve nets achieve organism-wide coordination through diverse mechanisms, including through-conduction, regionally autonomous ensembles, and volume transmission. They discuss how in jellyfish, specific sub-nets drive behaviours like umbrella infolding, and how computational models show that simple local interaction rules can generate complex, global activity patterns without the need for centralized command hubs. The authors highlight the ctenophore aboral nerve net as a coordination network that synchronizes balancer cilia, via specific synaptic targets.

These examples and themes are put together to illustrate that that the diffuse architecture of nerve nets is not merely an evolutionary stepping stone or a primitive remnant, but a convergent, highly evolved solution ideal for the locomotion and signalling requirements of radially symmetrical, soft-bodied animals.

Overall, the manuscript is free of major errors or inaccuracies and covers a lot of ground. Many of the figures are of high quality and informative. The literature is extensive but could benefit from a few additions (detail below). The main downside is that the writing is rather exhausting and disconnected. I sympathise with the challenge the authors have in synthesizing a large, disparate volume of information into a coherent article, but I think they could do a better job if they lead each section with more compelling questions that they then answer, using the literature and examples.

With careful restructuring and fixes to some of the writing (detail below) the article will make a useful addition, drawing together literature that is often overlooked in a prominent venue. But it needs to draw readers in and convince outsiders to this topic that there are compelling, big picture lessons that they can take away.

*We thank the reviewer for these comments. We have incorporated the specific suggestions and references and revised and restructured the text.*

## Main comments

The opening section “what is a nerve net” lays out the purpose of the article but does so vaguely. It would help to lay out the claims and contributions explicitly: for example, are we questioning the definition of a nerve net? Are we making claims about evolutionary origins or function? A pointed paragraph will let the reader know what to expect and allow the reader to evaluate whether the article delivers its promises.

In the next section the authors state that “A null hypothesis for nerve nets would posit a loose and disorderly organisation” why? What would this imply and why would deviation from it be informative? Has the claim they are random been made, and if so by whom? One could argue that simpler nervous systems would have more stereotypical organisation, with the wiring more constrained by a simple and rigid developmental program, for instance. The hypothesis seems to be a straw man, in other words, and its main purpose seems to be to point out that structure varies (but so what?). This should be motivated by more convincing and granular hypotheses. Doing so would give better structure and interpretability to the numerous paragraphs on pages 4-5, which currently reads as a rather exhausting list of facts.

*We have now rewritten and reorganised this section. We start with a very clear mathematical definition of a random graph and also cite examples from the literature where nerve nets are considered random. We then systematically test the null hypothesis of random organisation and reject it.*

The section on connectomics should cite more of the mainstream connectomics literature and methodologies (e.g. in C elegans, Drosophila, mouse….). It would be helpful to describe some of the challenges and limitations of the methodology, especially in applying it to new species. This section does not bring home any clear points, other than mentioning the existence of a connectome (from single/multiple specimens?) with a throwaway sentence at the end that doesn’t provide an insightful conclusion. The reader is left wondering what we learned from connectomics in these species and what we stand to learn.

*Discussing the techniques and challenges of vEM and connectomics is beyond the scope of the paper. We have now more clearly describe the insights from EM work and what we have learned from the new connectome resources.*

The section on Spread of activity (P7 onwards) would really benefit from diagrams showing modes of how activity propagates in the different systems mentioned.

*We agree, however, given the large variety of methods and scales, we did not find a good way to summarise these finding in a concise figure.*

The conclusion could do with something bolder. “Future directions” being to study nerve nets in more detail is not very compelling. The authors should try to consider what non-

specialist readers should take away from the article and how the study of nerve nets changes our thinking about neurophysiology, development, evolution and so on.

*We have now shortened the conclusions, removed the sections about 'more work is needed' and close with a bold outlook: "Nerve nets in combination with distributed sensory-integrative-pacemaker centres, such as in jellyfish, represent a fascinating organisational principle. This contrasts with a centralised single-command-centre architecture and may be more resilient to damage, hijacking or even disease. The distributed organisation of nerve nets could inspire improvements in diverse man-made systems, including computing, traffick, administration, or social media (the Fediverse vs. one centralised platform). If we stopped considering animals with nerve nets as 'primitive' we could start learning from them."*

## Minor comments/typos/grammar/clarification

Figure 1: would benefit from a (abbreviated!) phylogenetic tree to illustrate how diverged the species are. P1 "The other is theories" - pluralisation?

*Changed to: 'The other context is theories…'*

P1 "sometimes lump vastly different diffuse organisations as one character state" - clarify

*Changed to: 'These reconstructions are often very coarse-grained and simply assign to animal phyla either of two character states (nerve net, vs. centralised nervous system)'*

P3 "endodermal nets are very differently organised" - vague, clarify

*Changed to: 'The ectodermal and endodermal nerve nets of the small freshwater hydrozoan Hydra are also diffuse, although the ecto- and endodermal nets differ in anatomy and function. The ectodermal nerve net is synaptic but there are no or only few neuromuscular synapses in the endoderm [@keramidioti2024; @Zhang_2025]. The ecodermal nerve net, but not the endodermal net, activates during fast body-column contractions [@Dupre2017].'*

P3 "neuronal markers revealed a layered organisation" - tense

*Changed to: 'neuronal markers reveal a layered organisation'*

Figure 3: panels are too close together and text is too small in panels D and E

*We have revised Figure 3 as suggested.*

P8 "Electrical recordings from hydromedusae found that their nerve nets show mor regional autonomy than in scyphomedusae" - orphaned sentence, should be in a paragraph

*This sentence was integrated with the paragraph on calcium imaging in hydromedusae: 'Electrical recordings from hydromedusae found that their nerve nets show more regional autonomy than in scyphomedusae [@Passano_1965pacemakers;*

*@satterlie2002]. This regionalisation was recently confirmed by calcium imaging experiments [@Weissbourd2021; @Cunningham_2024Jellyfish].'*

P10 "The nerve net likely signals to the muscles by synapses, present between…" clarity - consider rewriting to "The nerve net likely signals to the muscles via synapses between…"

*Changed to: 'The nerve net likely signals to the muscles via synapses between the ectodermal net and muscles.'*

P16 (and throughout) "Simply put, being 'diffuse' is not a useful character" - I think you mean "characteristic". Consider substituting throughout the article as this confused me.

*Changed to: characteristic or trait, except when we write 'character state', as this is widely used in the literature*

P16 "perpetuated by ladder-thinking (instead of tree-thinking)" - I understand what you mean but some readers may benefit from a follow on sentence expanding on this metaphor.

*Explanation added: "They are in part perpetuated by ladder-thinking (i.e. reading phylogenies as ladders of progress [@Omland_2008Tree]) instead of branching trees) that is pervasive in the literature on cnidarians."*

## Referee #2:

The author's manuscript is a timely and exciting topic that discusses classical and recent findings about nerve nets. The review covers anatomical, developmental, physiological and evolutionary aspects over a range of different phyla that possess this type of nervous system. The authors conclude that nerve nets are diverse, often show a polarized organization and functional specialization. Nerve nets vary not only in anatomy but also in transmission architecture, using chemical synapses, gap junctions or syncytial fusions as well as volume transmission through neuropeptides as means of connecting neurons with each other and to sensor and effector cells. These findings put together, and in perspective, hint towards an extremely intricate and complex system of behavioral control and signal processing through nerve nets. While the topic and the papers covered here are certainly exciting and the review will be interesting for a broad readership, the manuscript is not well structured. Unfortunately, concepts and lines of thought, are mixed and make it very difficult to follow the ideas and text flow, specifically, if one is not an expert in the field. Animals from different phyla and subphyla are mixed and phylogenetic relationships are not considered with respect to the nerve net organization. Hypotheses are not presented logically and may even be opposed a few paragraphs later which is confusing. While I feel that this review will be beneficial to the field, I strongly recommend a rigorous editing Below please find some more detailed examples:

## Major concerns:

• The manuscript is not well structured, concepts and lines of thought, are mixed and make it very difficult to follow the ideas and text flow, specifically, if one is not an expert in the field.

*We have significantly restructure the text. We have also indicated when we write about concepts or specilations. We added a new heading “Nerve nets — conceptualisation” to initiate the discussion of random nets from the perspectice of graph theory. We also revised the language e.g. “This conceptualisation will help us to discuss…”*

• The text is too detailed for a review article, especially in some aspects (e.g. Ctenophore nerve net) and many passages read like summaries of original research rather than a review. Concretely, this means that non experts may have to read primary literature to understand large parts of the text. This is also prevalent later in the text in which the mathematical modeling part reads like a summary of the paper.

*We have rewritten and reorganised several sections of the text and removed some of the details about ctenophores and the mathematical models.*

• Animals from different phyla and subphyla are mixed and phylogenetic relationships are not considered with respect to the nerve net organization and other speculations. Generalizations might therefore better be used with care.

*We have added a phylogenetic overview to guide the readed. We refrain from explicit cladistic analysis, because of the sparse sampling and the lack of high-resolution studies from most animals. We also changed the text and are more careful about generalisations, see also detailed responses to specific comments below. When writing a paper we decided to organise the text by organisational principles and functional characteristics rather than phylogeny, also to avoid the temptation of a simplistic cladistic framework.*

• Some hypotheses (especially in the first part of the manuscript) are not presented logically and may even be opposed a few paragraphs later which is confusing. The concepts underlying the formulation of the hypothesis need rigorous work. This is especially important as there are not just nerve nets and brains but a variety of intermediate neuronal structures (which the authors discuss at the very end- it might be beneficial to consider restructuring the organization of the text).

*We have restructured the text and included a clear starting null hypothesis (a random graph). We also restructured several sections to make the presentation more logical. We decided to keep the presentation of more centralised structures in a later section, to keep the focus on nerve nets.*

• Definitions, Figures and Schemes are not presented logically, come several paragraphs later or are non-existent. For example diffuse, disorganized, random, disorderly and unpolarized are used without definition and interchangeably. The current text without clear definitions can be highly confusing and would benefit from an initial

explanation (definition) of the different terminology, followed by a consistent use of those words in their respective context.

*We have included clear definitions of ‘plexus’, ‘nerve net’, and ‘ganglion’ based on Richter_2010Invertebrate.*

• Many Figures are not mentioned at the respective text passages or at all (for some aspects of the figures (e.g. subfigures A,B…), while difficult concepts are not depicted in the figures.

*We have revised all figures and refer to all subpanels at the respective text passages.*

• The authors speculate on many aspects and often the speculations are not connected by structured analysis transferring observations and concepts between phyla, subphyla or species. This is particularly evident with respect to inference of physiological functions without solid molecular and cellular physiology present in many of the described species. The authors infer function across different species, from transcriptomics or anatomy. In my opinion this is not best practice, and such formulations should be labelled as theories or speculations that await further analysis.

*We have revised the text and removed some of the inferences and speculations. We however disagree that one should not or cannot infer function from transcriptomics (e.g. transmitters and their receptors, neuropeptides, sensory genes etc.) or anatomy (e.g. axons, dendrites, sensory modifications, synapses etc. – connectomics would not exist then).*

• English grammar, text composition, words and tense are incorrect throughout the text.

*We have revised the text based on the specific suggestions by the reviewer, see below.*

• Many passages cannot be understood without being familiar with the primary literature and are very detailed and complicated while elsewhere critical information is missing.

*We thank the reviewer for the many detailed and specific comments below that we tried to address fully.*

• I am not familiar with all literature, but some content is reflected incorrectly (see examples below) and these points are not reflected critically or appear to be selectively used to support arguments of the authors.

*Corrected, see below.*

• I highly recommend shortening the manuscript. Many of the later theories are discussed extensively elsewhere. Specific examples: -first paragraph: both species and definition seem confusing: many animals within the list possess ganglia like structures, such as Rhopalia, apical organs, nerve rings and neuronal concentrations e.g. hydra and anemones around foot and mouth region etc in which concentrations and polarity are found. Seems good to reconsider phrasing here. In addition, the authors make the very same point a few paragraphs later and discuss these points towards the end of the text.

*We have revised the text, and also added an additional paragraph that mentiones many of the more centralised examples the reviewer pointed out: "It is important to note that many animals with nerve nets can also possess more condensed neuronal structures, such as nerve rings or rhopalia in cnidarians, ganglionic concentrations in anemones, an anterior commissural brain in acoels, a dorsal and ventral cord in hemichordates, etc. Some of these examples of condensation within or alongside nerve nets will be discussed towards the end of the manuscript."*

"A null hypothesis for nerve nets would posit a loose and disorderly organisation." Do not understand this sentence, also as mentioned before: just by the fact that these animals possess structural and tissue organization, sensor and effector cells/tissues there is an inherent polarization to the nerve net- the authors must formulate their thoughts and the origin of this hypothesis more clearly. A few paragraphs later they state: "Nerve nets of a fully random organisation likely do not exist", thereby nullifying a theory whose origin was not clear to begin with.

*We have rephrased and clarified these sections and added the definition of a random graph. "A formal definition is lacking, therefore we will go back to graph theory to conceptualise…". The idea of random connectivity is widespread in the literature and textbooks, and several examples are cited in [@Satterlie_2011Do]. It is therefore a useful and valid starting point (null hypothesis) for our discussion, even if we concluded that such random nerve nets likely do not exist. The point of a null hypothesis is to be tested and falsified.*

-" In hemichordates - bilaterian animals with a diffuse nerve plexus and belonging to the ambulacraria": as mentioned previously- it would be good if there were some definitions, specifically in animals where neuronal structures are forming intermediate structures between NN and centralized aspects. Nerve plexus here means nerve net?

*We now cite the neuroanatomical glossary of Richter_2010Invertebrate and briefly define the terms plexus, ganglion and nerve net.*

-" Second, neurons within nerve nets show functional specialisations.": This is highly speculative as different transcripts and morphology must not necessarily equal different functionality. At the very minimum, the authors should support this claim with a functional study or reformulate this sentence.

*We disagree, different molecular profile and morphology more likely equal different functionality. We have now strenghtened this argument and mention that the transcriptionally different neuron types express e.g. different neuropeptides that suggest that they contribute differentially to chemical signalling networks. "These molecular and morphological differences likely equal different functionality. For example, some of the endodermal neurons are sensory, others neurosecretory or ganglion-like. Different neuron types also express different combination of neuropeptides indicating differential contributions to chemical signalling networks."*

-"In the ctenophore subepithelial nerve net (SNN), no anatomically polarity has been observed.": this paragraph is in the middle of neurite polarization. Might be better to reorganize this paragraph?

*We have changed this statement (there is in fact some level of polarity in the SNN as reported by Courtney_2022Characterization) and reorganised the paragraph. "Ctenophores possess a polygonial subepithelial nerve net (SNN) and a distinct aboral nerve net (ANN)…"*

-"However, this nerve net is also well organised with uniform tiling of the body.": To me, it is unclear what this sentence means.

*We removed this sentence and rewritten this paragraph to improve structure and clarity. "Ctenophores possess a polygonial subepithelial nerve net (SNN) and a distinct aboral nerve net (ANN)…*

-Under cellular organization, the authors state that the synapses are non-polarised and bidirectional. A few paragraphs later, the authors reverse this statement: "Fourth, synaptic connectivity in nerve nets is also non-random and follows clear structural rules andsynaptic polarity (with defined pre- and postsynaptic partners)."

*We have reorganised and reworded this section: "Fourth, connectivity in nerve nets is often non-random and is polarised. In Hydra, the nerve net forms many polarised synaptic contacts on postsynaptic partners [@Westfall1971].."*

-" tip-to-tip "neuronal handshakes"" I recommend to change this formulation for clarity, how is this not random? And what would be random? The definition comes several lines later: In Hydra, the endodermal nerve net of a small animal has been reconstructed. "This nerve net is formed of 20 neurons wherein neurons only connect to their immediate neighbours. Surprisingly, no classical chemical synapses could be identified in this dataset. Instead, neurites of adjacent neurons formed digiform tips, which interdigitated with partner neurites. These structures - termed neural handshakes - were devoid of vesicles indicating that they do not function as classical synapses. It remains to be determined if these handshakes contain gap junctions, as seems likely (Keramidioti et al, 2024a)."

*We have reorganised this paragraph. "In Hydra,there are many polarised synaptic contacts formed by the nerve net on postsynaptic partners [@Westfall1971]. In the endodermal nerve net,synapses are lacking, but neurons form connections via specialised digiform tips that interdigitate with partner neurites. These structures — termed "neuronal handshakes" — only form between immediate neighbours[@Zhang_2025]."*

*The Hydra network is a lattice with adjacent cells connecting. It would be random if any cell could connect to any other cell (not only to its neighbours) with equal probability.*

-" The bidirectional synapses observed in cnidarians (Anderson & Schwab, 1981) and also ctenophores (Westfall, 1996) constitute a special case that likely occurs where non-directional transmission is an advantage, such as within the swimming-motor nerve net of jellyfish. Such nonpolarised transmission may not represent the default or ancestral state." This statement is highly speculative, and the point is reversed as mentioned elsewhere

*We removed this statement and reworded the section.*

-” In a pioneering study, Peteya was able to infer the overall synaptic connectivity of the ectodermal nervenet of the anthozoan cerianthid Ceriantheopsis americanus Peteya (1973).” This is not vEM- the text flow here as also in many other places is extremely misleading

*We have rearranged and rewritten this section and indicated that Peteya did not do vEM but analysed single sections*

“Instead, neurites of adjacent neurons formed digiform tips, which interdigitated with partner neurites.” What does this mean? “Digiform” is not an English word- if anatomical description, this needs to be explained.

*Changed to ‘protrusions’*

-the description of the ctenophore ANN is very difficult to comprehend

*We have simplified and reorganised this section.*

-” This spread is nonpolarised as first shown by Romanes in a series of elegant incision experiments (Romanes, 1885) (Figure 2). When he incised the umbrella in complex patterns, he found that the transmission of the excitation could pass around the incisions, showing diffuse and nonpolarised spread.” This statement should be toned down as later Mackie, Passano and others showed that hydroids have neuroid conduction via epithelia which can explain the results of the incisions, see also: “Neuroid conduction and the evolution of conducting tissues” by G.O. Mackie 1970

*Thank you for pointing this out, we added this information to the paragraph: “However, later Mackie, Passano and others showed that hydroids have neuroid conduction via epithelia which can explain the results of the incisions [@mackie1970; @passano1973behavioral; @Kasssimon1973TRANSMITTINGSI].”*

-“In the body wall, the singular and extensive syncytial network may mediate fast and spatially extensive conduction.” This is highly speculative and very unclear to me how this would work physiologically.

*We have rephrased this as a question: ” How far and how fast activity spreads in these syncytial networks will require the development of live imaging approaches in ctenophores.”*

-“These likely form diffusion barriers and a slow the spread of activity, potentially limiting activation to local sectors.” This statement is speculative and potentially wrong- nodes of Ranvier are also constricted and potentiate spread of activity. As mentioned elsewhere, it would be desirable not to infer physiological function from anatomy.

*We have reformulated this as a question for future studies: “How these different anatomies influence the spread of activity will require further studies.”*

-“One classical example is provided by Hernandez-Nicaise et al. (1980), who showed that the giant smooth musclecells of Beroë ovata receive dense chemical synaptic input from the nerve net and are capable of generating sodium-dependent action potentials (Hercnandez-Nicaise & Amsellem, 1980).” This Paper actually states that the AP is

carried by both sodium and calcium, see also Liebeskind et al 2011- Ctenophores most likely possess only calcium selective Nav channels.

*We have corrected this sentence and now mention calcium potentials*

-"In Nematostella, body length expands and contracts depending on feeding state, and neuron numbers in both the ectodermal and endodermal nets change reversibly in parallel. When the animal is bisected, the regenerating polyp reconstructs a nerve net with fewer neurons, yet the network scales appropriately to the reduced body size and fully restores behaviors such as feeding, contraction, and touch responses (Havrilak et al, 2017)." Here newer literature might be incorporated: Gavgani et al 2025 also show that the nervous system is relevant to guide recovery.

*We have incorporated this reference here: "The nervous system also regenerates after an almost complete non-surgical, chemogenetic ablation [@Mazloumi_Gavgani_2025Ectopic]. Amputation experiments with such nerve-free polyps also revealed an unexpected function of the nervous system in defining axial polarity during whole-body regeneration."*

-"Instead, it reflects the ability of the syncytial nerve net to establish electrophysiological coupling and a newly continuous tissue, allowing coordinated motor output across the chimeric individual." In my opinion, electrophysiological is both ambivalent and speculative here: this could rely on other mechanisms such as volume transmission, however, physiological proof is required to make any such statement.

*We removed the term 'electrophysiological' and reworded the sentence. We think that volume transmission would be too slow for the coupled fast contructions. "Instead, it suggests that the syncytial nerve net is able to establish coupling possibly by fusion, allowing coordinated motor output across the chimeric individual."*

-"These ancestors were isopotential and the sensory, control and effector layers were combined in a single cell or an electrically coupled colony of cells. The" Not all protists are isopotential, see for example G.O Mackies 1970s Neuroid conduction and the evolution of conducting tissues: Noctiluca. As mentioned elsewhere, best not to generalize these statements, there may be much out there that we do not yet know or understand. It is also unlikely that the fused choanoflagellates mentioned below are isopotential.

*We have removed the mention of isopotential cells.*

-" In general, a pacemaker circuit would require some feedback and multi-neuron ensemble, which suggests that projections and synapses came before pacemaker activity." It is unclear to me how the authors come to this conclusion- pacemakers can be found on a molecular level too.

*This statement has been removed, and we now ask the question how rhythmic behaviours could be regulated in synapse-less sponges and placozoans, synapse-less animals that also show evidence of rhythmic activity (e.g. contractions).*

## Figures:

Fig1: -Figure and text are not in harmony: Here organized and polarized NN are shown, with concentrations of neurons into sensory organs, while the authors discuss the opposite in the text

*Figure 1 shows examples of various nerve nets across animal groups. We refer to this figure now in the general introductory section, before we conceptualise random nerve nets (Figure 2)*

Fig2: In connection with Figure 1 and logic error in the text: It is very unclear where the hypothesis (or facts) of a disorganized nerve net come from. This theory would gain strength if the authors had some examples of where or why they assume that the net is “random and unpolarized”,

*We have extended and clarified this section, see above.*

Figure 2 is only mentioned once in the text. 2E) looks similar to Weissbourd et al 2021 - may be appropriate to cite the paper here.

*We now mention Figure 2 in different parts of the text and refer to individual subpanels. We cited Weissbourd et al in the legend.*

Figure3: Is not connected to the text, specifically for the explanation of the ctenophore NN this will be very helpful

*We have redesigned this figure (now Figure 4) and connected it better to the text. We also re-wrote the section on the ctenophore ANN.*

Figure 4 and 5: subparts are not mentioned or connected to the text.

*Now corrected.*

## Minor aspects:

-check citations, some are annotated wrong in the text e.g. (Havrilak et al, 2025) is actually 2017

*We cite two papers by Havrilak et al., one from 2017 and one from 2025*

-abbreviations are not introduced (e.g. AP anterior posterior)

*The AP abbreviation was removed (only two mentions), others are introduced; subepithelial nerve net (SNN); aboral nerve net (ANN).*

-speculations about function and physiology must be clearly labelled as such

*We have removed/reworded some of the speculations and either formulated the as questions or labelled speculations/inferences about function as such e.g. ‘it may’, ‘We hypothesize that’.*

-best not to use generalizations like “in all other nerve nets” given the small number of organisms in which those have been sampled and analyzed

*Changed to: ‘In contrast to cnidarian and bilaterian nerve nets’*

-spreading of electrical activity, electrical coupling: speculative

*Revised or indicated as speculative with ‘may’.*

-Volume transmission: introduce concept

*We have added an explanatory sentence: ” During volume transmission neuropeptides or other transmitters (e.g. monoamines) are released along the neurites of source neurons and act on nearby receptor-expressing target cells.”*

-the five aspects the authors are describing could be perceived more structured with subheadings

*We have included sub-heading to describe the four aspects (changed five to four), e.g. ‘Cellular organisation — distribution of neuronal somata’*

-development- more and newer citations for cnidarian nerve centralization

*We now also cite Zhong_2026Morphological showing the ganglionic organisation of the oral nerve ring in Nematostella.*

-“The aboral nerve net synapses on ciliated balancer cells and highspeed imaging of balancer cilia revealed coordinated regulation of beat frequency, arrests and re-beats.” Citation missing

*added*

Dear Prof. Jékely,

We have now received the comments of the two original referees regarding your revision. While in general they acknowledge that the topic is very timely and interesting and that you revised the manuscript according to their previous comments, they also raise several remaining comments that call for your attention. It would be fantastic if you can try to accommodate their comments and revise your review accordingly.

When preparing your letter of response to the referees' comments, please bear in mind that this will form part of the Review Process File, and will therefore be available online to the community. For more details on our Transparent Editorial Process, please review our Editorial Policies page: https://link.springer.com/partners/embo-press/editorial-policies

We generally allow three months as standard revision time, but I would appreciate it if you can revise and submit your manuscript at your earliest convenience.

Yours sincerely,

Yehu Moran
Academic Editor
The EMBO Journal

Instructions for preparing your revised manuscript:

Read our guidance for manuscript revisions and related editorial policies: https://link.springer.com/journal/44318/submission-guidelines#cms-Revised-submissions

Please also check that the title and abstract of the manuscript are brief, yet explicit, even to non-specialists.

When assembling figures, please refer to our figure preparation guideline in order to ensure proper formatting and readability in print as well as on screen:
https://media.springernature.com/original/springer-cms/rest/v1/content/27825798/data/v1

IMPORTANT: When you send the revision we will require
- a point-by-point response to the referees' comments, with a detailed description of the changes made (as a word file).
- a word file of the manuscript text.
- individual production quality figure files (one file per figure)
- a complete author checklist
- Expanded View files (replacing Supplementary Information)
- a Reagents and Tools Table as part of the Methods section

Please remember: Digital image enhancement is acceptable practice, as long as it accurately represents the original data and conforms to community standards. If a figure has been subjected to significant electronic manipulation, this must be noted in the figure legend or in the 'Methods' section. The editors reserve the right to request original versions of figures and the original images that were used to assemble the figure.

We realize that it is difficult to revise to a specific deadline. In the interest of protecting the conceptual advance provided by the work, we recommend a revision within 3 months (31st May 2026). Please discuss the revision progress ahead of this time with the editor if you require more time to complete the revisions. Use the link below to submit your revision:

https://emboj.msubmit.net/cgi-bin/main.plex

------------------------------------------------

Referee #1:

The revisions have improved the clarity and structure of the manuscript. The clear layout in the introduction now prepares the reader for what is to come and why the numerous facts are relevant. The writing is still a bit of a list of facts and rather tiring to

read. It is a shame about the figure on activity propagation, but that's up to the authors.

The authors completely missed my point about the random net hypothesis. I wasn't asking for a mathematical definition of a random net. I was asking why the assumption of randomness is biologically relevant: it seems to be a straw man with one reference citation tied to it. Repeating it at the start of each section, only to immediately refute it with a deluge of evidence is a rather tedious exercise. There should be stronger and more interesting biological questions to motivate the curation of these facts, and it would help if the authors could think of them. Otherwise it reads as though all this work was done to disprove something that most biologists likely didn't believe in the first place.

The authors can take or leave my comments. The article could be published as is, or they could attempt to elevate it while they have the opportunity. In any case I don't need to look over a further revision as I feel this is an editor's call.

Referee #2:

The text reads better after the revision. The null hypothesis is embedded in the text and connected to the figures. The definitions at the beginning are helpful to clarify reoccurring concepts throughout the text. Shortening the suggested passages has been advantageous.

Some minor points:

-the authors might want to consider being less repetitive in their wording (e.g. may, could)
-shorten text more?
-check for grammar, punctuation, and typos

# Response to reviewers - second round

## Referee #1:

The revisions have improved the clarity and structure of the manuscript. The clear layout in the introduction now prepares the reader for what is to come and why the numerous facts are relevant. The writing is still a bit of a list of facts and rather tiring to read. It is a shame about the figure on activity propagation, but that's up to the authors.

The authors completely missed my point about the random net hypothesis. I wasn't asking for a mathematical definition of a random net. I was asking why the assumption of randomness is biologically relevant: it seems to be a straw man with one reference citation tied to it. Repeating it at the start of each section, only to immediately refute it with a deluge of evidence is a rather tedious exercise. There should be stronger and more interesting biological questions to motivate the curation of these facts, and it would help if the authors could think of them. Otherwise it reads as though all this work was done to disprove something that most biologists likely didn't believe in the first place.

The authors can take or leave my comments. The article could be published as is, or they could attempt to elevate it while they have the opportunity. In any case I don't need to look over a further revision as I feel this is an editor's call.

Response: We thank the reviewer for these comments. - We have now added further panels to Figure 2 to better illustrate the different modes of activity propagation within nerve nets. - We have revised the text so that it does not seem like we are dealing with a straw man. We still think that many biologists believe that nerve nets are in general rather disorganised and (close to) random. We have now provided further references and quotations. In light of this, we think that providing a clear quantitative null hypothesis is a good conceptual framework. Such a framework could encourage future more quantitative assessments of nerve-net connectivity and structure (e.g. in future connectomics analyses). "A widespread perception of nerve nets is that they are diffuse networks of neurons with random projections and connectivity. According to the definition of Hejnol and Rentzsch"a basic nerve net consists of an irregular arrangement of neurites" for the formation of which the "random outgrowth of neuronal processes in a permissive environment might be sufficient" (Hejnol & Rentzsch, 2015). Further paraphrased quotes assuming a random or 'diffuse' organisation are cited in (Satterlie, 2011)."

## Referee #2:

The text reads better after the revision. The null hypothesis is embedded in the text and connected to the figures. The definitions at the beginning are helpful to clarify reoccurring concepts throughout the text. Shortening the suggested passages has been advantageous.

Some minor points:

-the authors might want to consider being less repetitive in their wording (e.g. may, could) -shorten text more? -check for grammar, punctuation, and typos

Response: We have further shortened the text and did further editing of typos and grammar.

Hejnol A & Rentzsch F (2015) Neural nets. *Current Biology* 25: R782–R786

Satterlie RA (2011) Do jellyfish have central nervous systems? *Journal of Experimental Biology* 214: 1215–1223

## 2nd Revision - Editorial Decision 7th Jul 2026

Dear Prof. Jékely,

Thank you for submitting your manuscript to The EMBO Journal. It has now been evaluated by the two original reviewers. While both are supportive of the revised version, one reviewer has identified a minor issue with one of the figures. Please address this point and resubmit the manuscript at your earliest convenience, so that we can proceed with formal acceptance.

Thank you again for submitting this fascinating review to The EMBO Journal. I look forward to receiving your final revision.

Yours sincerely,

Yehu Moran
Academic Editor
The EMBO Journal

Instructions for preparing your revised manuscript:

Read our guidance for manuscript revisions and related editorial policies: https://link.springer.com/journal/44318/submission-guidelines#cms-Revised-submissions

Please also check that the title and abstract of the manuscript are brief, yet explicit, even to non-specialists.

When assembling figures, please refer to our figure preparation guideline in order to ensure proper formatting and readability in print as well as on screen:
https://media.springernature.com/original/springer-cms/rest/v1/content/27825798/data/v1

IMPORTANT: When you send the revision we will require
- a point-by-point response to the referees' comments, with a detailed description of the changes made (as a word file).
- a word file of the manuscript text.
- individual production quality figure files (one file per figure)
- a complete author checklist
- Expanded View files (replacing Supplementary Information)
- a Reagents and Tools Table as part of the Methods section

Please remember: Digital image enhancement is acceptable practice, as long as it accurately represents the original data and conforms to community standards. If a figure has been subjected to significant electronic manipulation, this must be noted in the figure legend or in the 'Methods' section. The editors reserve the right to request original versions of figures and the original images that were used to assemble the figure.

We realize that it is difficult to revise to a specific deadline. In the interest of protecting the conceptual advance provided by the work, we recommend a revision within 3 months (5th Oct 2026). Please discuss the revision progress ahead of this time with the editor if you require more time to complete the revisions. Use the link below to submit your revision:

https://emboj.msubmit.net/cgi-bin/main.plex

------------------------------------------------

Referee #1:

The revision addresses my previous comments and is in good shape.

Referee #2:

Figures 2J and 2K are overlapping

Other than that my concerns have been addressed.

All editorial and formatting issues were resolved by the authors.
